# Integrin Activation Enables Sensitive Detection of Functional CD4^+^ and CD8^+^ T Cells: Application to Characterize SARS-CoV-2 Immunity

**DOI:** 10.3389/fimmu.2021.626308

**Published:** 2021-03-29

**Authors:** Anna Schöllhorn, Juliane Schuhmacher, Luciana Besedovsky, Rolf Fendel, Anja T. R. Jensen, Stefan Stevanović, Tanja Lange, Hans-Georg Rammensee, Jan Born, Cécile Gouttefangeas, Stoyan Dimitrov

**Affiliations:** ^1^Department of Immunology, Institute for Cell Biology, University of Tübingen, Tübingen, Germany; ^2^Institute of Medical Psychology and Behavioral Neurobiology, University of Tübingen, Tübingen, Germany; ^3^Institute of Tropical Medicine, University of Tübingen, Tübingen, Germany; ^4^Department of Immunology and Microbiology, University of Copenhagen, Copenhagen, Denmark; ^5^German Cancer Consortium (DKTK) and German Cancer Research Center (DKFZ) Partner Site Tübingen, Tübingen, Germany; ^6^Cluster of Excellence iFIT (EXC2180) “Image-Guided and Functionally Instructed Tumor Therapies, ” University of Tübingen, Tübingen, Germany; ^7^Department of Rheumatology and Clinical Immunology, University of Lübeck, Lübeck, Germany; ^8^German Center for Diabetes Research (DZD), Tübingen, Germany; ^9^Institute for Diabetes Research and Metabolic Diseases of the Helmholtz Center Munich at the University of Tübingen (IDM), Tübingen, Germany

**Keywords:** integrin activation, antigen specificity, CD4^+^ T cells, CD8^+^ T cells, SARS-CoV-2

## Abstract

We have previously shown that conformational change in the β_2_-integrin is a very early activation marker that can be detected with fluorescent multimers of its ligand intercellular adhesion molecule (ICAM)-1 for rapid assessment of antigen-specific CD8^+^ T cells. In this study, we describe a modified protocol of this assay for sensitive detection of functional antigen-specific CD4^+^ T cells using a monoclonal antibody (clone m24 Ab) specific for the open, high-affinity conformation of the β_2_-integrin. The kinetics of β_2_-integrin activation was different on CD4^+^ and CD8^+^ T cells (several hours vs. few minutes, respectively); however, m24 Ab readily stained both cell types 4–6 h after antigen stimulation. With this protocol, we were able to monitor *ex vivo* effector and memory CD4^+^ and CD8^+^ T cells specific for severe acute respiratory syndrome coronavirus 2 (SARS-CoV-2), cytomegalovirus (CMV), Epstein–Barr virus (EBV), and hepatitis B virus (HBV) in whole blood or cryopreserved peripheral blood mononuclear cells (PBMCs) of infected or vaccinated individuals. By costaining β_2_-integrin with m24 and CD154 Abs, we assessed extremely low frequencies of polyfunctional CD4^+^ T cell responses. The novel assay used in this study allows very sensitive and simultaneous screening of both CD4^+^ and CD8^+^ T cell reactivities, with versatile applicability in clinical and vaccination studies.

## Introduction

One of the first events that can be detected upon T cell receptor (TCR)-mediated stimulation of T cells is the activation of membrane-bound β_2_-integrins. This activation involves a conformational change (from the bent, inactive form to the open, high-affinity conformation) and a clustering of integrin molecules on the cell membrane. Activated integrins are essential for ligand binding [e.g., the binding of the β_2_-integrin leukocyte function-associated antigen 1 (LFA-1) to intercellular adhesion molecule (ICAM)-1] for immunological synapse formation and downstream cell functions.

In cytotoxic effector CD8^+^ T cells (CTLs), β_2_-integrins participate in the interaction with target cells (e.g., virus-infected or cancer cells), facilitating downstream functions, such as directed granzyme, perforin, and cytokines secretion and, ultimately, cell killing (1–4). A good correlate to these CD8^+^ T cell functions is, therefore, an integrin conformational change, as it can be detected earlier than the conventional flow cytometry cytotoxic markers, such as CD107a and tumor necrosis factor (TNF). Using fluorescent multimers of ICAM-1, we have previously shown that effector CD8^+^ T lymphocytes can be specifically and very rapidly detected upon antigen-specific T cell stimulation. We observed that the binding of ICAM-1 multimers to β_2_-integrins on CD8^+^ T cells identifies functional effectors that will later degranulate and produce effector cytokines. In addition to being a quick readout (approximately after 4–20 min stimulation in most cases) based on the use of a single detecting reagent, the assay preserves cell viability and can be used for the enrichment of antigen-responding CD8^+^ T cells for an immediate analysis or for a post *in vitro* expansion analysis (5, 6).

CD4^+^ T helper cells primarily interact with major histocompatibility complex (MHC)-class II antigen-presenting cells and exert fundamentally different functions in comparison to CD8^+^ T cells. The main effector molecules of effector CD4^+^ T cells are cytokines and costimulatory receptors, such as CD154 (CD40L), all of which are routinely used for measuring CD4^+^ T cell immune responses (7, 8). The formation of immunological (of CD4^+^ T cells) and cytotoxic (of CD8^+^ T cells) synapses differs substantially. The former takes hours to be established and is stable once formed, whereas the contacts formed by CD8^+^ T cells are brief and CD8^+^ T cells can establish multiple cytotoxic synapses over a short period of time (9, 10). The kinetics of β_2_-integrin activation on CD4^+^ T cells is not known so far, but based on the properties of synapse formation, we can expect it to be slower than that of CD8^+^ T cells. Still, the integrin activation should occur earlier than the expression of cytokines and CD154 and, thereby, might provide a fast and easily detectable surface marker to be used for monitoring and isolation of CD4^+^ T cells.

In the present study, we describe a modified protocol of the ICAM-1 multimer assay, which is also taking advantage of β_2_-integrin activation on T cells but was adapted for sensitive detection of antigen-specific CD4^+^ effectors. Instead of ICAM-1 multimers, a monoclonal antibody (Ab) specific for the open, high-affinity conformation of the β_2_-integrin (Ab clone m24 (referred hereafter as m24 Ab); (11, 12)), combined with the ethylenediaminetetraacetic acid (EDTA) treatment to disrupt the cell conglomerates formed during CD4^+^ T cell activation, was used. The assay can be used for simultaneous screening of both CD4^+^ and CD8^+^ T cell reactivities and should be useful for a high-throughput detection of antigen-specific T lymphocytes, epitope mapping studies, and the monitoring of clinical trials. Staining with m24 Ab together with CD154 enables sensitive and accurate detection of extremely low frequencies of polyfunctional CD4^+^ T cells. The study presents examples of antigen-specific CD4^+^ and CD8^+^ T cell detection in whole blood (WB) or cryopreserved peripheral blood mononuclear cells (PBMCs) obtained from cytomegalovirus (CMV)- and Epstein–Barr virus (EBV)-infected volunteers and from hepatitis B virus (HBV) vaccinees. In addition, severe acute respiratory syndrome coronavirus 2 (SARS-CoV-2)-directed T cells were readily detected in convalescents who have previously been tested positive for the virus and in an individual who received a SARS-CoV-2 peptide vaccine.

## Materials and Methods

### Study Subjects and Blood Samples

First, we obtained either PBMC concentrates or Na-heparinized blood from healthy individuals. Following donors were selected for the study: one person with detectable CD4^+^ T cells binding to the HLA-DRB1*11 multimer refolded with the CMV peptide HPTFTSQYRIQGKLE; two HLA-A*02^+^ people with detectable CD8^+^ T cells binding to the HLA-A*02:01 multimer refolded with the CMV NLVPMVATV; one HLA-A*02^+^ person with detectable CD8^+^ T cells binding to the HLA-A*02:01 multimer refolded with the EBV BRLF1 peptide YVLDHLIVV and a CD4^+^ T cell reactivity to an in house-made HLA-class II peptide-pool containing nine CMV, EBV and Flu-derived epitopes. Two individuals vaccinated with hepatitis B vaccine (Engerix-B, GlaxoSmithKline, London, UK) approximately 10 years ago were also included. PBMCs were isolated by cell density centrifugation (Biocoll, Biochrom AG, Berlin, Germany), were washed twice in phosphate-buffered saline (PBS) without Ca^2+^/Mg^2+^, and were frozen in aliquots in 90% heat-inactivated fetal calf serum (Thermo Fisher Scientific, Waltham, MA, USA) + 10% dimethyl sulfoxide (DMSO) (Merck, Darmstadt, Germany).

Na-heparinized blood was also obtained from three convalescent persons who had been recently diagnosed with the SARS-CoV-2 infection (either by PCR testing after nasopharyngeal swab or by serological Ab testing) and additionally from four uninfected control donors. Na-heparin plasma was isolated by centrifugation. Anti-CoV-2 Ab testing was performed with the SARS-CoV-2 ELISA from EUROIMMUN, (Lübeck, Germany). Tests were specifically conducted for detecting spike protein domain 1 (S1)-specific immunoglobulin G (IgG) and nucleocapsid (N)-specific IgG. ELISA was performed according to the instructions of the manufacturer. All three individuals developed serum IgG against S1 and/or N at the time of T cell testing, whereas unexposed control donors did not reveal any IgG reaction, as shown in **Figures 7**, **8**. All studies were approved by the Ethics Committee of the University of Tübingen (approvals 156/2012B01, 713/2018BO2, and 256/2020BO2), and participants provided written informed consent.

A self-experimenting healthy volunteer was vaccinated with SARS-CoV-2-derived peptides, with details presented in a study by Rammensee et al. [(13) Preprint]. Briefly, the donor received subcutaneous injections of synthetic peptides emulsified in Montanide™ ISA (Seppic SA, Paris, France) together with the TLR 1/2 ligand XS15 as an adjuvant. This experimental vaccine contained, among others, the four following HLA-class II peptides: CMV/YQE (which had been already applied in 2017), CoV/IGY, CoV/ASA, and CoV/FYV (see “Peptides, stimuli, and pMHC tetramers” section). According to the results of an interferon-gamma (IFN-γ) ELISpot experiment performed ~3 weeks after vaccination, all four peptides had induced a T cell response *in vivo* [(13) Preprint]. For the experiment shown in **Figure 9**, blood was obtained ~6 weeks after vaccination.

Characteristics of all donors (*n* = 11 healthy individuals without a history of CoV-2 infection and *n* = 3 CoV-2 convalescents) are summarized in Table 1.

**Table 1:** Demographic characteristics of the participants.

**Participants #**	**Age**	**Gender**	**Tested specificities**	**Corresponding figures**
#1	60	Male	CMV, SEB	1, 3, 5, 12, S1, S3, S5
#2	32	Male	CMV, SEB	2, 3, 4, S2
#3	30	Male	HBV, SEB	3, 4, 6, 12, S3, S5
#4	49	Male	HBV	3, 12, S4
#5	31	Male	SEB	3, 4
#6	67	Male	CoV-2	9, 12
#7	53	Female	MHCII peptides, EBV	10
CoV-2 #1	51	Male	CoV-2	7, 8, 12
CoV-2 #2	63	Female	CoV-2	7, 8, 12
CoV-2 #3	50	Female	CoV-2	7, 8, 12
UD #1	27	Female	CoV-2	7, 8
UD #2	29	Female	CoV-2	7, 8
UD #3	22	Female	CoV-2	7, 8
UD #4	53	Female	CoV-2	7, 8

### Peptides, Stimuli, and pMHC Tetramers

For antigen-specific stimulation, we used the following synthetic peptides, representing known T cell epitopes derived from virus-associated antigens: for CD4^+^ T cell stimulation, HLA-DRB1^*^11:01-restricted HPTFTSQYRIQGKLE peptide from CMV pp65, aa 366–380 (CMV/HPT, donor #1) and a mix of nine HLA-class II binders from various proteins of CMV (*n* = 2), EBV (*n* = 6), and flu (*n* = 1) viruses (donor #7); for CD8^+^ T cell stimulation, the HLA-A^*^02-restricted NLVPMVATV peptide from CMV pp65, aa 495–503 (CMV/NLV, donor #2), and the HLA-A^*^02-restricted YVLDHLIVV peptide from EBV BRLF1, aa 109–117 (EBV/YVL, donor #7).

HLA-class II peptides used for experimental CoV-2 immunization were CMV pp65, aa 510–524 YQEFFWDANDIYRIF (CMV/YQE); SARS-CoV-2 nucleocapsid (N), aa 83–98 IGYYRRATRRIRGGD (CoV-2/IGY) and aa 311–326 ASAFFGMSRIGMEVT (CoV-2/ASA); and SARS-CoV-2 envelope (Env), aa 56–70 FYVYSRVKNLNSSRV (CoV-2/FYV, donor #6). All peptides were synthesized and dissolved as previously described (purity ≥80%) (14). We also used pools of overlapping 15-mer peptides spanning the entire HBV/Env (donors #3 and #4), SARS-CoV-2/Prot N, or SARS-CoV-2/Prot M or covering the immunodominant sequence domains of SARS-CoV-2/Prot S for activation. The HBV/Env peptide pool (JPT, Berlin, Germany) was dissolved in 100% DMSO, whereas all SARS-CoV-2 pools (Miltenyi, Bergisch Gladbach, Germany) in 10% DMSO. All were aliquoted and kept at −80°C until further use. Staphylococcus enterotoxin B (SEB; Sigma-Aldrich, Darmstadt, Germany) was solved in PBS without Ca^2+^/Mg^2+^ and frozen at −20°C in 1 mg/ml aliquots. As a negative control, we used cells in the medium alone (control experiments showed that the background activation was not significantly different between cells cultured alone, or in the presence of mock DMSO/water, or of control HLA-class II binding peptides derived from self or the HIV, data not shown).

We produced biotinylated pHLA-A^*^02:01 monomers (CMV A^*^02/NLV, donor #2) in-house by conventional refolding, as previously described (15). We generated fluorescent pHLA-A^*^02:01 tetramers by coincubating streptavidin-phycoerythrin (PE) or -allophycocyanin (APC) (BioLegend, San Diego, CA, USA and Thermo Fisher Scientific, Waltham, MA, USA) at a 1 (streptavidin):4 (pHLA-A2 monomer) molar ratio. Multimers were aliquoted and stored at −80°C in a Tris-buffered saline (TBS) buffer containing 16% glycerol (15). PE-labeled DRB1^*^11:01 CMV/HPT tetramer was kindly provided by the NIH Tetramer Core Facility (Atlanta, GA, USA) and stored at 4°C until use (donor #1).

### Production of Human ICAM-1 Multimers

Soluble fluorescent ICAM-1 complexes were produced as described previously by Dimitrov et al. (5). We generated fluorescent ICAM-1-Fc/anti-Fc multimeric complexes by co-incubating 200 μg/ml recombinant human ICAM-1-Fc [produced and purified as previously described (16) or bought from BioLegend (San Diego, CA, USA; cat. number 552906)] with 3.41 × diluted polyclonal anti-human Fc-PE F(ab′)_2_ fragments (Jackson ImmunoResearch Labs Inc., West Grove, PA, USA) at 4°C for 24 h while slightly rotating (15 rpm). For example, to prepare 100 μl of 200 μg/ml ICAM-1 multimers using ICAM-1-Fc from BioLegend (San Diego, CA, USA), we mixed 24.4 μl ICAM-1-Fc (4.1 × dilution, final concentration 200 μg/ml) with 29.3 μl Fc-PE F(ab′)_2_ fragments (3.41 × dilution) and 46.3 μl sterile PBS. The stock solutions of the anti-human Fc-PE F(ab′)_2_ fragments, which came lyophilized, were prepared according to the recommendation of the manufacturer (diluted in 1 ml Milli-Q water, Merck Millipore, Darmstadt, Germany). We confirm from experience the stability of the multimeric ICAM-1 complexes for at least 2 months when stored at 4°C. For longer storage, ICAM-1 multimers can be kept at −80°C, but not at −20°C, as this increases significantly the background staining.

### Cell Stimulation, m24 Ab, or ICAM-1 Multimer Stainings

We used fresh heparinized blood or frozen PBMCs for the assays. WB was used within 2 h after collection. Cryopreserved PBMCs were thawed, washed, and rested overnight in T cell medium (TCM) [IMDM (Lonza, Basel, Switzerland) with 10% heat-inactivated human serum, 100 U/ml penicillin/100 μg/ml streptomycin (Sigma-Aldrich, St. Louis, MO, USA), and 50 μM β-mercaptoethanol (Merck)] containing 1 μg/ml DNAse I (Sigma-Aldrich, St. Louis, MO, USA), and then, they were washed and counted on the Neubauer chamber (Thermofisher, Waltham, MA, USA) before stimulation and staining.

We stimulated WB (up to 1 ml per test) or PBMCs at 2 × 10^6^ cells/ml (1000 μl/test) or 1 × 10^7^ cells/ml (200 μl/test) in TCM, for the indicated times at 37°C (water bath or incubator) in 5 ml or 15 ml Falcon tubes (BD Biosciences, Heidelberg, Germany) with the following peptides: 4 μg/ml of the single peptides CMV/HPT, CMV/HQE, CoV/IGY, CoV/ASA, CoV/FYV, EBV/GLC; a pool of EBV/YVL and 9 HLA-class II binders with 4 μg/ml per peptide; 2 μg/ml of the peptide pools HBV/Env, SARS-CoV-2/ S, CoV-2/ N, CoV-2/M; or 4 μg/ml SEB.

After stimulation, we left the tubes at room temperature (RT) for 1–4 min and stained the cells at RT with a pretitrated amount (1:80 to 1:200) of the m24 Ab clone (BioLegend, San Diego, CA, USA) labeled with PE. After 15-min incubation, EDTA was added for another 10 min at RT at a final concentration of 4 mM. Whenever indicated, the cells were also stained with HLA-A^*^02/pMHC multimers (0.6 μg/ml).

For alternative staining with ICAM-1 multimers after stimulation, we incubated the cells with ICAM-1 multimers for 4 min at 37°C and left the tubes at RT for 5 min. Whenever indicated, the cells were incubated with EDTA for 10 min at RT at a final concentration of 4 mM or stained with pA^*^02 multimers (0.6 μg/ml).

After m24 Ab or ICAM-1 multimers staining, we fixed the samples and lysed erythrocytes with the fluorescence-activated cell sorting (FACS)-Lysing Solution (BD Biosciences, San Jose, CA, USA) for WB or fixed the samples with the 1.5% Fix Solution (Polysciences, Inc., Warrington, PA, USA) for PBMCs, followed by washing with PBS/0.5% bovine serum albumin (BSA)/0.1% sodium azide. After centrifugation, we stained the cells for surface markers in order to identify CD4^+^ T cells or CD8^+^ T cells with Abs CD3-PerCP-Cy5.5, CD4-BV421, and CD8-BV605/APC in PBS/0.5% BSA/0.1% sodium azide for 15 min at RT. Cells were washed once and fixed in PBS/0.5% BSA/0.5% formaldehyde. All Abs used were from BioLegend (San Diego, CA, USA).

For MHC-class II tetramer staining (**Figure 3**), PBMCs were incubated with 18 μg/ml DRB1^*^11:01 CMV/HPT tetramer for 3 h at 37°C diluted in 200 μl PBS/0.5% BSA (Sigma-Aldrich, St. Louis, MO, USA), washed once, and then Ab CD4-FITC (in-house production), CD8-PE-Cy7 (Beckman Coulter, Brea, CA, USA), and Zombie Aqua (BioLegend, San Diego, CA, USA) were added in 50 μl PBS/0.5% BSA for another 15 min. The cells were washed once with PBS/0.5% BSA/0.1% sodium azide and fixed in PBS/0.5% BSA/1% formaldehyde.

### m24 Ab Staining Combined to Intracellular Cytokine Staining

For the assessment of intracellular CD154 and cytokine expression, we stimulated up to 1 ml WB or PBMCs at 37°C (2 × 10^6^ or 1 × 10^7^ cells/ml in TCM) for the indicated times and with the indicated peptides and concentrations in the presence of 10 μg/ml brefeldin A (Sigma-Aldrich, St. Louis, MO, USA) and 5 μg/ml monensin (BD Biosciences, San Jose, CA, USA). After stimulation, we left the tubes at RT for 1–4 min; then, extracellular staining with m24-PE Ab, together with Zombie Aqua staining when indicated, was performed for 15 min at RT, followed by incubation with 4 mM EDTA for 10 min at RT. Samples were fixed and erythrocytes were lysed with the FACS-Lysing Solution, followed by washing with PBS/0.5% BSA/0.1% sodium azide. CD3-PerCP-Cy5.5, CD4-BV421, and CD8-APC-Cy7 Abs were added in PBS/0.5% BSA/0.1% sodium azide for 15 min at RT. After one washing step in PBS/0.5% BSA/0.1% sodium azide, the cells were then permeabilized for 20 min at RT with the FACS-Perm2 Solution (BD Biosciences, San Jose, CA, USA) and washed with PBS/0.5% BSA/0.1% sodium azide, followed by intracellular staining with IFN-γ-FITC, IL-2-PE-Cy7, TNF-BV605, and CD154-APC Abs in PBS/0.5% BSA/0.1% sodium azide for 30 min at RT. The cells were washed once with PBS/0.5% BSA/0.1% sodium azide and fixed in PBS/0.5% BSA/0.5% formaldehyde. All Abs used in the study were purchased from BioLegend (San Diego, CA, USA).

### Flow Cytometry and Data Analysis

**Figure 1:**
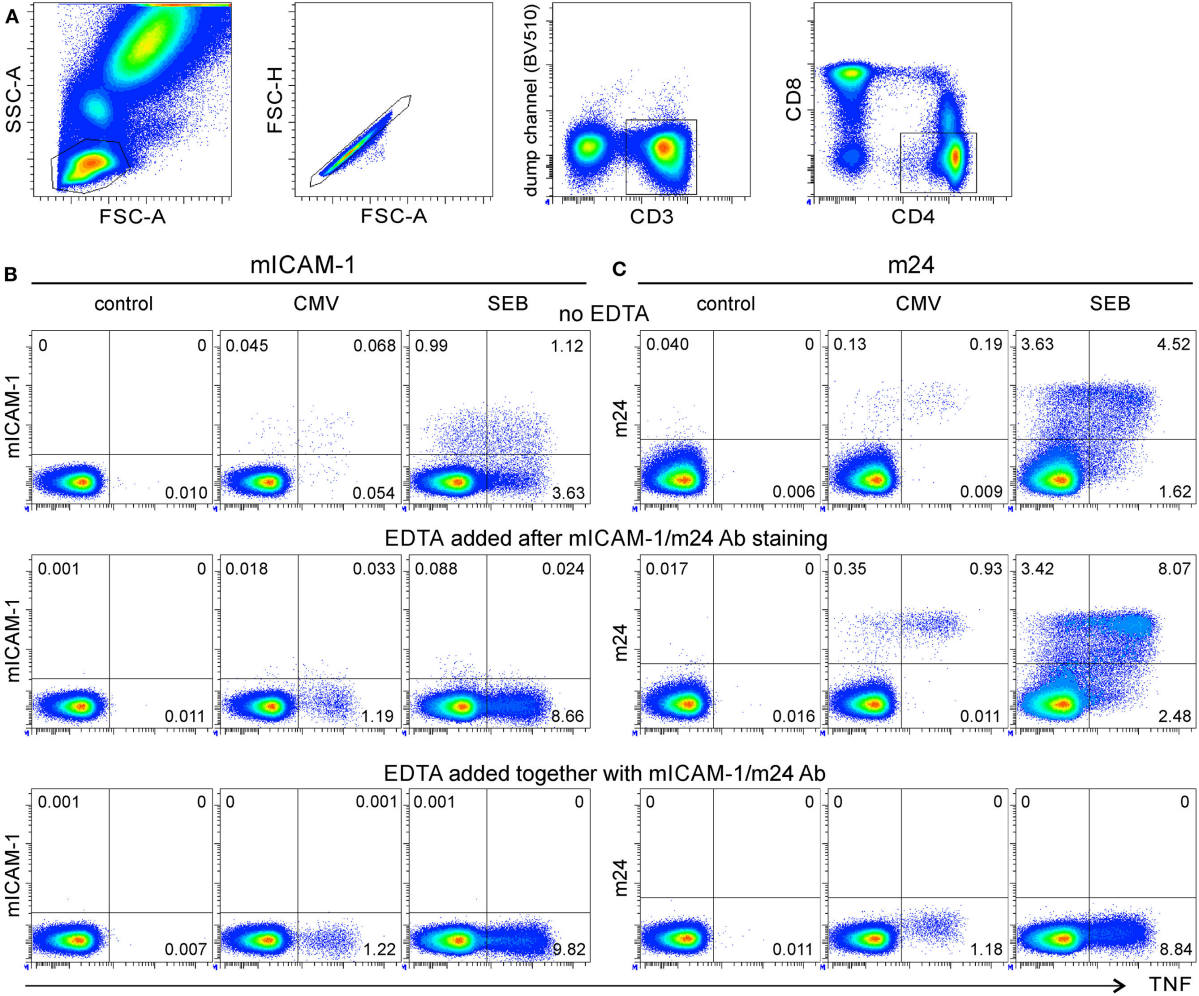
m24 Antibody (Ab) staining of activated β_2_-integrins followed by ethylenediaminetetraacetic acid (EDTA) treatment can be used to detect antigen-specific CD4^+^ T cells. Assessment of whole blood (WB) antigen-specific CD4^+^ T cells by staining of activated β_2_-integrins. **(A)** Gating strategy. From left to right, the lymphocyte gate, the FSC-A/H duplet exclusion, the gating of CD3^+^ events followed by CD4^+^ events. **(B,C)** Intercellular adhesion molecule-1 (ICAM-1) multimer and m24 Ab stainings under different EDTA treatment. WB from a donor having a large number of CMV/HPT-specific CD4^+^ T cells was cultured without stimulus (left, control), with the CMV/HPT peptide (middle), or with Staphylococcus enterotoxin B (SEB) (right) in the presence of brefeldin A and monensin. After 4 h, cells were either stained with multimers of ICAM-1 (mICAM-1) for 4 min **(B)** or with m24 Ab for 15 min **(C)**, without (top), or with EDTA addition after (middle), or at the time (bottom) of the staining. The cells were subsequently labeled with CD3, CD4, and CD8 Abs and for the intracellular expression of tumor necrosis factor (TNF) to identify CMV-specific CD4^+^ T cells. Numbers indicate frequencies among CD4^+^ T cells in %.

**Figure 2:**
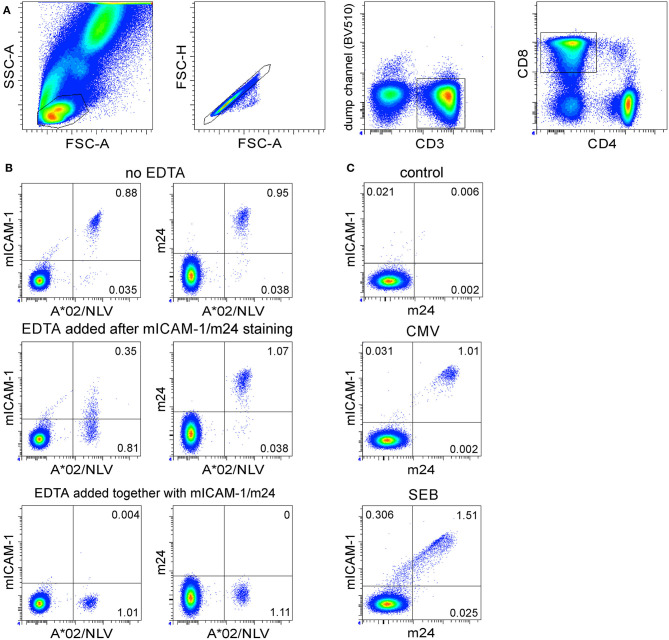
The antigen-specific β_2_-integrin activation on CD8^+^ T cells can be visualized by either ICAM-1 multimers (without EDTA) or by m24 Ab with or without EDTA treatment. Assessment of WB antigen-specific CD8^+^ T cells by staining of activated β_2_-integrins. **(A)** Gating strategy. From left to right, the lymphocyte gate, the FSC-A/H duplet exclusion, the gating of CD3^+^ events, followed by CD8^+^ events. **(B)** The WB from a selected HLA-A*02^+^ donor was incubated with the NLV peptide for 16 min, then either not treated (top) or treated with EDTA after (middle) or at the time of (bottom) the staining with multimers of ICAM-1 or with m24 Ab. Cells were simultaneously stained with A*02/NLV tetramers and thereafter with CD3, CD4, and CD8 Abs. **(C)** Staining with m24 Ab and ICAM-1 multimers together, without EDTA treatment. Numbers indicate frequencies among CD8^+^ T cells in %.

We acquired the data on an LSRFortessa cell analyzer (BD Biosciences, San Jose, CA, USA); analyses were performed with either the FACS DiVa version 8.0 software or the Flow Jo 10.6.2 (BD Biosciences, Heidelberg, Germany) software. We collected between 50,000 and 400,000 CD4^+^ or CD8^+^ events for the antigen-specific assays. Gating strategies are shown in Figures 1, 2 and in Supplementary Figures 1, 2, 5. Results are presented as the percentage of cells within the parent populations.

## Results

### Antigen-Specific CD4^+^ T Cells Can Be Best Visualized With an Ab Specific for Activated β_2_-Integrins

We have previously shown that antigen-specific CD8^+^ T cells are successfully detected by staining of activated β_2_-integrins with fluorescent ICAM-1 multimers (5). We, therefore, investigated whether ICAM-1 multimers binding could also be used as a marker for the detection of antigen-specific CD4^+^ T cells. We selected a healthy donor with a high frequency of *ex vivo* detectable CD4^+^ T cells specific for the HPTFTSQYRIQGKLE epitope derived from the CMV pp65 protein (CMV/HPT). The WB cells were incubated with the CMV/HPT peptide, were incubated with SEB, or remained unstimulated for 4 h, and ICAM-1 multimers were added for the final 4 min of activation. Blood cells were further processed for the detection of intracellular TNF production (gating strategy is shown in Figure 1A). CMV/HPT- and SEB-stimulated cells showed only modest staining for both TNF and activated integrins (Figure 1B, top). The result was unexpected since the frequency of CMV/HPT-reactive (TNF^+^) CD4^+^ T cells was found to be ~1.2% in prescreening experiments of this donor. To check whether the antigen-specific CD4^+^ T cells had aggregated during the activation, and were therefore excluded from the lymphocyte gate, we treated WB with 4 mM EDTA after (Figure 1B, middle), or at the time of (Figure 1B, bottom), ICAM-1 multimer staining. With EDTA, much higher frequencies of CMV/HPT-specific or SEB-reacting TNF^+^ CD4^+^ T cells were detected (~1.2% and 8.7% and 1.2% and 9.8%, respectively). However, these TNF^+^ cells were mostly ICAM-1 multimer^neg^, most probably because EDTA reversed (when used after the staining) or prevented (when used at the time of staining) ICAM-1 multimers binding to activated β_2_-integrins by chelating Mg^2+^ and Ca^2+^ ions required for β_2_-integrin conformational change and for stable interaction with ICAM-1 multimers. To confirm that the stimulation leads to an aggregation of antigen-specific CD4^+^ T cells, which can be disrupted by EDTA treatment, we analyzed the distribution of TNF^+^ CD4^+^ T cells within the lymphocyte gate (FSC-A/SSC-A) or within singlet cells (FSC-A/FSC-H). Without EDTA treatment, 15% of the CMV/HPT-specific and 42% of the SEB-responding TNF^+^ cells were within the lymphocyte or singlet gates, whereas EDTA treatment increased these numbers to 84–88% (Supplementary Figures 1A–D).

Because ICAM-1 multimers staining was not suitable for labeling activated β_2_-integrins on antigen-specific CD4^+^ T lymphocytes, we next tested staining of the cells with a monoclonal Ab that binds specifically to the extended/open high-affinity, but not to the resting, unactivated conformation of β_2_-integrins (m24 Ab). The WB cells from the same donor were either stimulated with the CMV/HPT peptide or with SEB or remained unstimulated for 4 h, and were then stained with m24 Ab for 15 min, followed by the detection of intracellular TNF. The unstimulated cells showed very low staining with m24 or anti-TNF Ab. Significant numbers of TNF^+^ CD4^+^ T cells were detected after the stimulation with CMV/HPT (0.2%) and SEB (6.1%), most of them being m24^+^ (Figure 1C, top); however, these were much less than when ICAM-1 multimers and EDTA were used. Addition of EDTA after (Figure 1C, middle), or at the time of (Figure 1C, bottom), m24 Ab staining improved TNF detection to expected frequencies (~1 and 9% for CMV/HPT and SEB, respectively). When EDTA was added after m24 Ab staining, the TNF-producing cells were essentially m24^+^, indicating that once the Ab is bound, EDTA does not reverse the binding, as it does for ICAM-1 multimers. When EDTA was added at the time of m24 Ab staining, the cells were m24^neg^, showing that EDTA blocked m24 Ab binding to activated β_2_-integrins. Without EDTA treatment, 18% of the CMV/HPT-specific and 50% of the SEB-stimulated TNF^+^ cells were within the lymphocyte or singlet gates, whereas EDTA treatment increased these numbers to 74–86% (Supplementary Figures 1E–H). Hence, m24 Ab staining of activated β_2_-integrins, followed by EDTA treatment, can be used to detect antigen-specific CD4^+^ T cells.

### Antigen-Specific β_2_-Integrin Activation on CD8^+^ T Cells Can Be Visualized by Either ICAM-1 Multimers or m24 Ab Staining

In a previous study, antigen-specific CD8^+^ T cells were detected with ICAM-1 multimeric complexes (without EDTA) (5). We, therefore, tested whether the staining of activated CD8^+^ T cells with m24 Ab would be comparable to that obtained with ICAM-1 multimers and to which extent EDTA influences detection. We selected an HLA-A^*^02^+^ donor with a high frequency of CD8^+^ T cells specific for the immunodominant epitope CMV pp65 NLVPMVATV (NLV). The WB cells were stimulated with the CMV/NLV peptide for 16 min. ICAM-1 multimers and HLA-A^*^02 (referred hereafter as A^*^02) tetramers refolded with the CMV/NLV peptide were added for the final 4 min of the activation (5); alternatively, m24 Ab and A^*^02/NLV tetramers were added after the stimulation, and the cells were incubated for 15 min. The gating strategy is shown in Figure 2A. The combination of A^*^02/NLV and ICAM-1 multimers identified 0.92% A^*^02/NLV^+^ CD8^+^ T cells (from which nearly all, i.e., 0.88%, were ICAM-1 multimer^+^, Figure 2B, top left), whereas the combination of A^*^02/NLV and m24 Ab identified 0.99% A^*^02/NLV^+^ CD8^+^ T cells (0.95% were m24^+^, Figure 2B, top right). The treatment with EDTA after ICAM-1 multimer or m24 Ab staining only marginally increased the frequency of tetramer^+^ cells (1.1%, Figure 2B, middle). Similar to what we had observed for CD4^+^ T cells, staining of activated integrins on CD8^+^ T cells was largely lost when EDTA was used after the staining with ICAM-1 multimers but not after the staining with m24 Ab. When EDTA was added together with ICAM-1 multimers or m24 Ab staining, the cells did not stain with either of the reagents (Figure 2B, bottom). To confirm that the stimulation did not aggregate the antigen-specific CD8^+^ T cells even without EDTA treatment, we analyzed the distribution of A^*^02/NLV^+^ CD8^+^ T cells on FSC-A/SSC-A and FSC-A/FSC-H plots (Supplementary Figures 2B,C, top). Without EDTA treatment, 80–81% of the CMV/NLV^+^ cells were within the lymphocyte or singlet gates, whereas EDTA treatment slightly increased these numbers to 91–93% (Supplementary Figures 2B,C, bottom).

Later, we assessed whether m24 Ab and ICAM-1 multimer stainings are interchangeable. The WB cells from the same donor were either stimulated with the CMV/NLV peptide or with SEB or remained unstimulated for 16 min; then, the cells were stained with ICAM-1 multimers and m24 Ab. Double stainings confirmed that both ICAM-1 multimers and m24 Ab essentially identify the same cells (Figure 2C).

Hence, antigen-specific β_2_-integrin activation on singlet CD8^+^ T lymphocytes can be visualized by either ICAM-1 multimers (without EDTA) or by m24 Ab with or without EDTA treatment, but for simultaneous detection of CD4^+^ and CD8^+^ T cells, m24 Ab and EDTA treatment should be combined.

### Kinetics of β_2_-Integrin Activation and m24 Ab Binding Is Different for CD4^+^ and CD8^+^ T Cells

**Figure 3:**
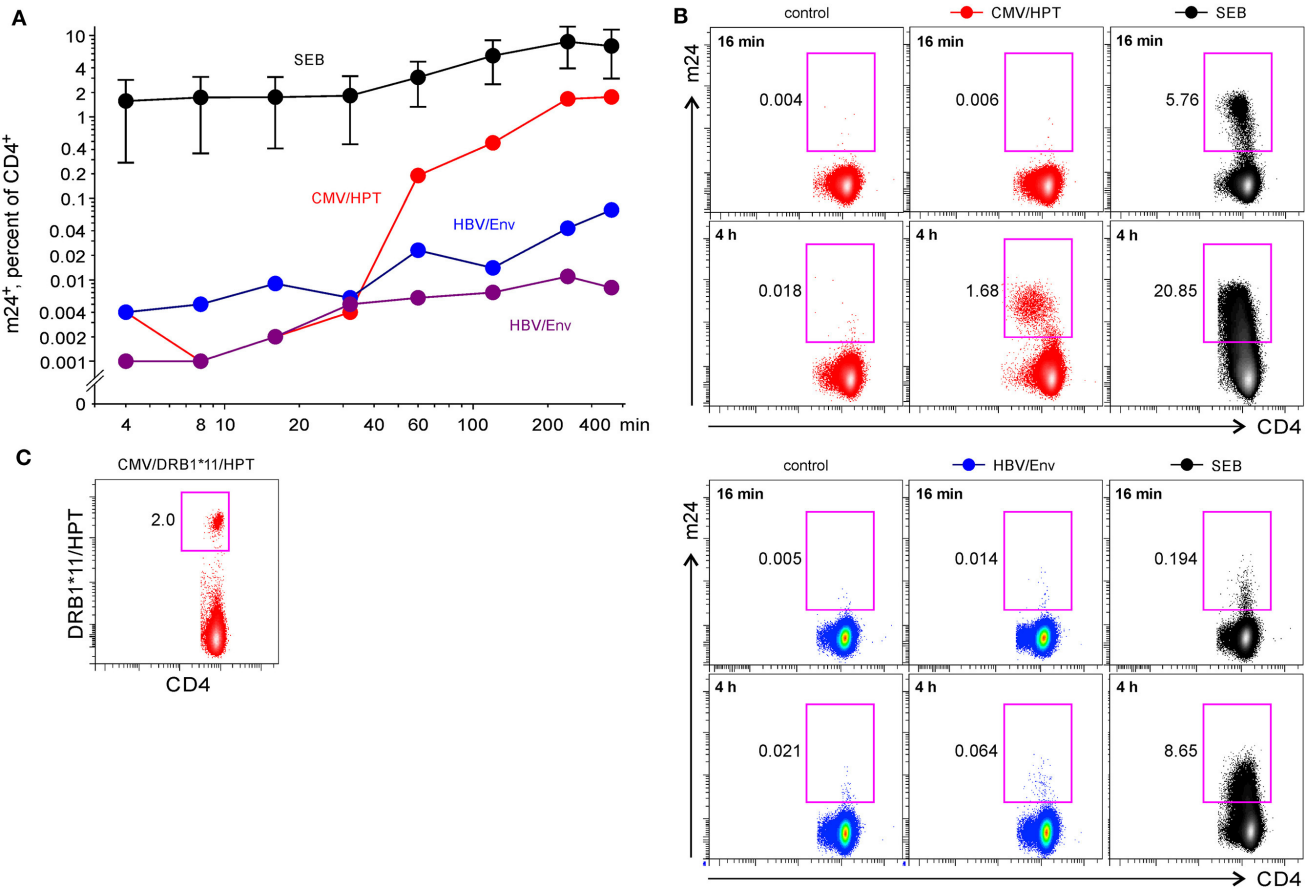
The flow cytometry assessment of antigen-specific CD4^+^ WB T cells by staining of activated β_2_-integrins with m24 Ab. The cells were stained with m24 Ab, treated with EDTA afterward, and then stained with CD3, CD4, and CD8 Abs, gating strategy is displayed in Figure 1A. **(A)** The time course of m24 Ab staining following incubation of WB cells with SEB (black, four donors, presented as mean ± SEM), CMV/HPT peptide (red, one donor), or HBV/Env pool of overlapping peptides (blue and purple, two donors) for 4, 8, 16, 32, 60, 120, 240, or 360 min. Background from the relevant unstimulated sample was subtracted. **(B)** Examples of m24 Ab staining obtained after 16 or 240 min, i.e., 4 h without stimulation (control) or in the presence of matched peptides or SEB of the donor with a CMV/HPT CD4^+^ reactivity (top panel). One of the two HBV vaccinees (bottom panel). **(C)** DRB1*11/HPT multimer staining was performed without CMV/HPT peptide stimulation and was obtained from the same donor as in **(B)** (top panel). Numbers indicate frequencies among CD4^+^ T cells in %.

**Figure 4:**
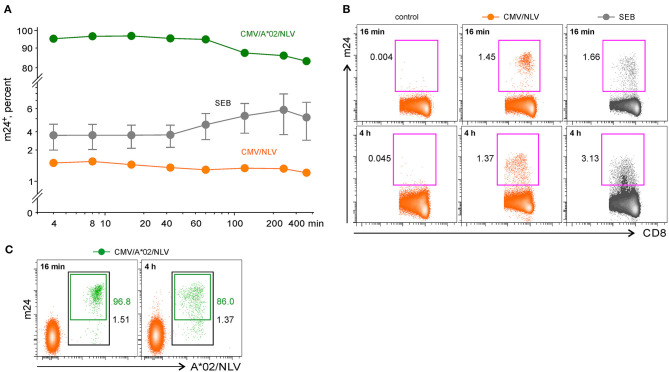
The flow cytometry assessment of antigen-specific CD8^+^ WB T cells by staining activated β_2_-integrins with m24 Ab. Cells were stained with m24 Ab, treated with EDTA afterward, and then stained with CD3, CD4, and CD8 Abs; gating strategy is displayed in Figure 2A. Percentages of m24^+^ cells among total CD8^+^ T cells are indicated. **(A)** The time course of m24 Ab staining following incubation of WB cells with SEB (gray, three donors, presented as mean ± SEM), or CMV/NLV peptide (orange, one donor; green, the same donor, but additionally stained with A*02/NLV multimers), for 4, 8, 16, 32, 60, 120, 240, or 360 min. Background from the relevant unstimulated sample was subtracted. Numbers indicate frequencies among CD8^+^ T cells in %. **(B)** Examples of m24 Ab staining obtained after 16 or 240 min, i.e., 4 h without stimulation (control), in the presence of CMV/NLV peptide, or after stimulation with SEB. **(C)** Combined m24 Ab and CMV/NLV tetramer staining of the CMV-specific CD8^+^ T cells. Percentages of A*02/NLV^+^ within the CD8^+^ subset and of m24^+^ within the A*02/NLV^+^ cells are indicated in black and green, respectively.

To determine the optimal duration for cell stimulation, we performed time courses of β_2_-integrin activation and m24 Ab binding on CD4^+^ and CD8^+^ T cells. The WB from one to four selected donors was stimulated with various antigens as indicated or remained unstimulated. Blood cells were harvested after different incubation times, followed by m24 Ab staining plus EDTA treatment. The kinetics is shown as % m24^+^ in Figure 3A (for CD4^+^ T cells) or in Figure 4A (for CD8^+^ T cells), representative examples are in Figures 3B,C (for CD4^+^ T cells) and Figures 4B,C (for CD8^+^ T cells).

A short-term activation with the CMV/HPT epitope in one donor induced only a weak m24 Ab staining on CD4^+^ T cells, whereas T cell response peaked after 4–6 h of stimulation (Figures 3A,B top). Costaining with HLA-DRB1^*^11 CMV/HPT tetramers and m24 Ab was not feasible because of strong TCR downregulation after peptide stimulation; however, we found comparable frequencies of specific cells with both methods [2% DRB1^*^11/HPT^+^ (Figure 3C) vs. 1.76% m24^+^ CD4^+^ T cells at the maximum of response after 6 h of stimulation with the CMV/HPT peptide]. Reactivity to overlapping 15-mer peptides (HBV/Env) in two HBV-vaccinated donors also reached a maximum between 4 and 6 h (Figures 3A,B bottom). For all four tested donors, the percentage of m24^+^ CD4^+^ T cells increased earlier after SEB stimulation but peaked after 4–6 h (Figures 3A,B). All unstimulated cells showed weak staining with m24 Ab (0.01–0.05%).

In contrast to antigen-specific CD4^+^ T lymphocytes, and as we previously observed with ICAM-1 multimers staining (5), responding CMV/NLV^+^ CD8^+^ T cells were very rapidly detected (immediate peak response of m24 Ab staining within only 4–16 min, Figure 4A). After prolonged activation with the CMV/NLV epitope (>1 h), the percentage of m24^+^ cells diminished only slightly (1.45 vs. 1.37% after 16 min or 4 h of stimulation, respectively, with a more substantial decrease found in the intensity, Figure 4B), in contrast to the strong decline we had observed when using ICAM-1 multimers (5). To confirm that m24 Ab selectively identifies antigen-specific T cells, we costained cells from the CMV-reactive HLA-A^*^02^+^ donor with A^*^02/NLV tetramers and m24 Ab. The majority of NLV-specific CD8^+^ T cells could be detected by m24 Ab binding, with maximal staining achieved within 4–16 min of activation (Figures 4A,C, 97% of double-stained cells after 16 min of stimulation). For CD8^+^ T cells, the frequency of m24^+^ cells after SEB stimulation peaked at 4 min in one case, whereas for the other two donors, it reached the maximum at 4 h, but with decreased staining intensity (Figures 4A,B). All unstimulated cells showed weak staining with m24 Ab (<0.01 to 0.05%).

We conclude that m24 Ab identifies antigen-responding CD4^+^ T cells, but in contrast to CD8^+^ T cells, several hours of stimulation are required to activate β_2_-integrins.

### m24 Ab Binding Identifies Functional Antigen-Specific CD4^+^ T Cells

**Figure 5:**
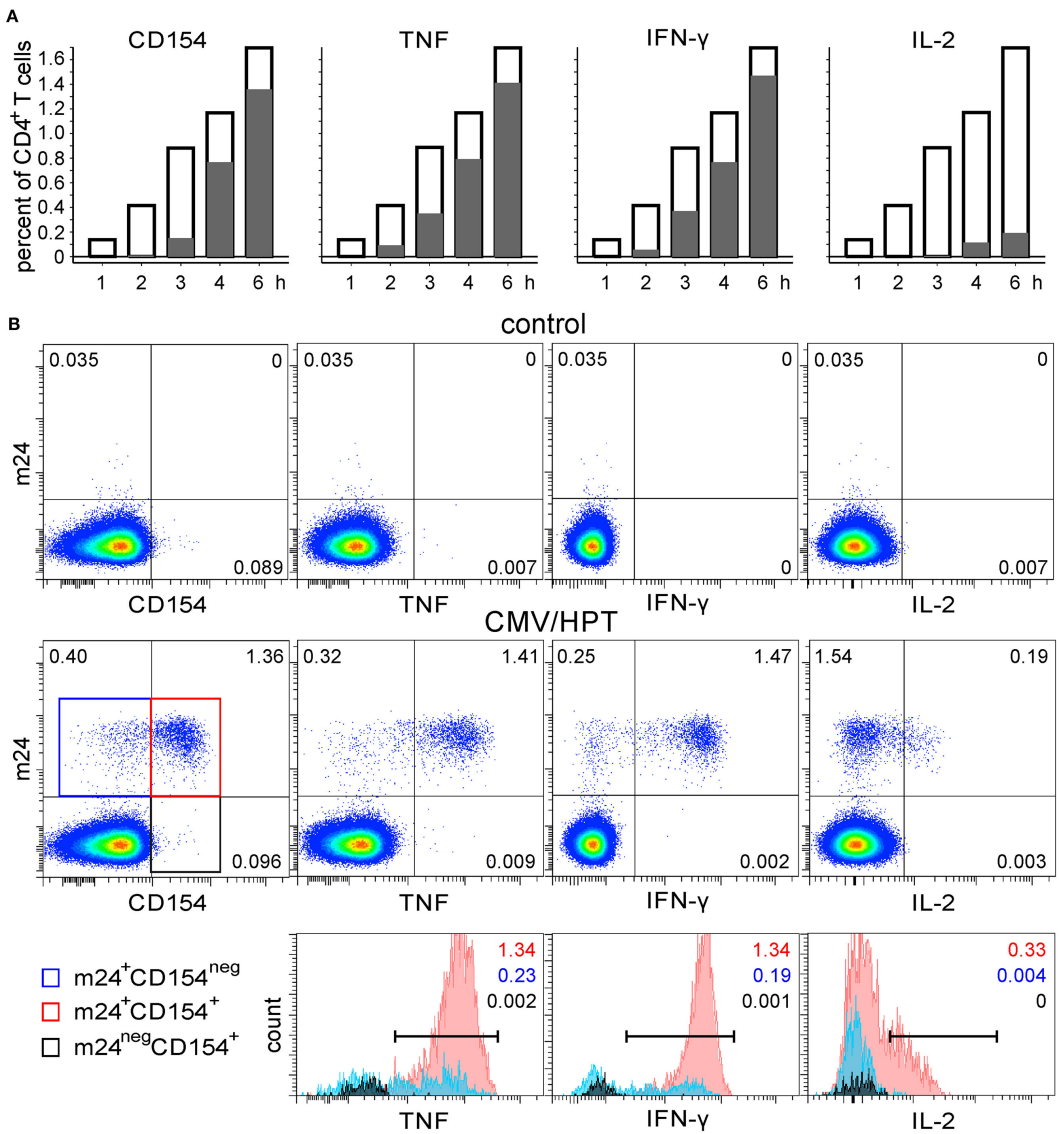
The functional profile of CMV-specific m24^+^ CD4^+^ WB T cells. WB of a DRB1*11^+^ donor was stimulated with CMV/HPT peptide. **(A)** Frequencies of m24 Ab staining, CD154, and cytokine expression within CD4^+^ T cells are shown as bar graphs (at the indicated times and after subtraction of the background assessed in the control test); empty bars represent total m24^+^ cells, positive gray bars represent marker^+^ (CD154, TNF, IFN-γ, or IL-2) m24^+^ CD4^+^ T cells, whereas negative gray bars (practically not visible) are marker^+^m24^neg^ CD4^+^ T cells. **(B)** Staining was obtained after 6 h without stimulation (control, top) or in the presence of CMV/HPT (middle). Numbers on density plots indicate frequencies among CD4^+^ T cells. m24^+^ CD154^neg^, m24^+^ CD154^+^, and m24^neg^ CD154^+^ subsets were gated (middle panel: blue, red, and black frames, respectively) and further displayed as histograms according to their TNF, IFN-γ, and IL-2 expression (bottom). Numbers on histograms indicate frequencies of the respective color-coded population among CD4^+^ T cells (markers are shown). Gating strategy is as shown in Figure 1A.

We next tested whether m24 Ab binding correlates with the functionality of antigen-specific CD4^+^ T cells. The WB from the DRB1^*^11^+^ donor was stimulated with the CMV/HPT peptide, was stimulated with SEB, or remained unstimulated. Blood cells were harvested after 1, 2, 3, 4, or 6 h, followed by incubation with m24 Ab plus EDTA treatment and staining for intracellular CD154 and cytokine (TNF, IFN-γ, and IL-2) upregulation. Results of the CMV/HPT stimulation are shown in Figure 5A, and density plots after 6 h of incubation without or with CMV/HPT are shown in Figure 5B, top and middle, respectively. m24 Ab binding was the earliest event to be detected (after 1–2 h of stimulation, Figure 5A, opened bars), and upregulation of CD154 and cytokines was exclusively confined to the m24^+^ cell subset [positive (marker^+^ m24^+^ cells) vs. negative (marker^+^ m24^neg^ cells), Figure 5A, gray bars]. Almost all m24^+^ cells (85%) expressed CD154, TNF, or IFN-γ at 6 h. Moreover, cytokine-producing cells were mainly m24^+^ CD154^+^. Only 15%, 12%, and 1% of the TNF-, IFN-γ-, or IL-2-producing cells did not express CD154, and virtually none were m24^neg^ (Figure 5B bottom). Some single stained m24^+^ or CD154^+^ events, but no double-stained m24^+^ CD154^+^ events, were observed in unstimulated cells (Figure 5B top, left). Comparable results were obtained for the SEB-stimulated cells in this individual, with a strong association between m24 Ab staining and TNF or IFN-γ production; still, a fraction of CD154^+^ or IL-2^+^ cells were m24^neg^; however, we found that CD154^+^ m24^neg^ cells were predominantly also cytokine^neg^ (Supplementary Figures 3A,B).

**Figure 6:**
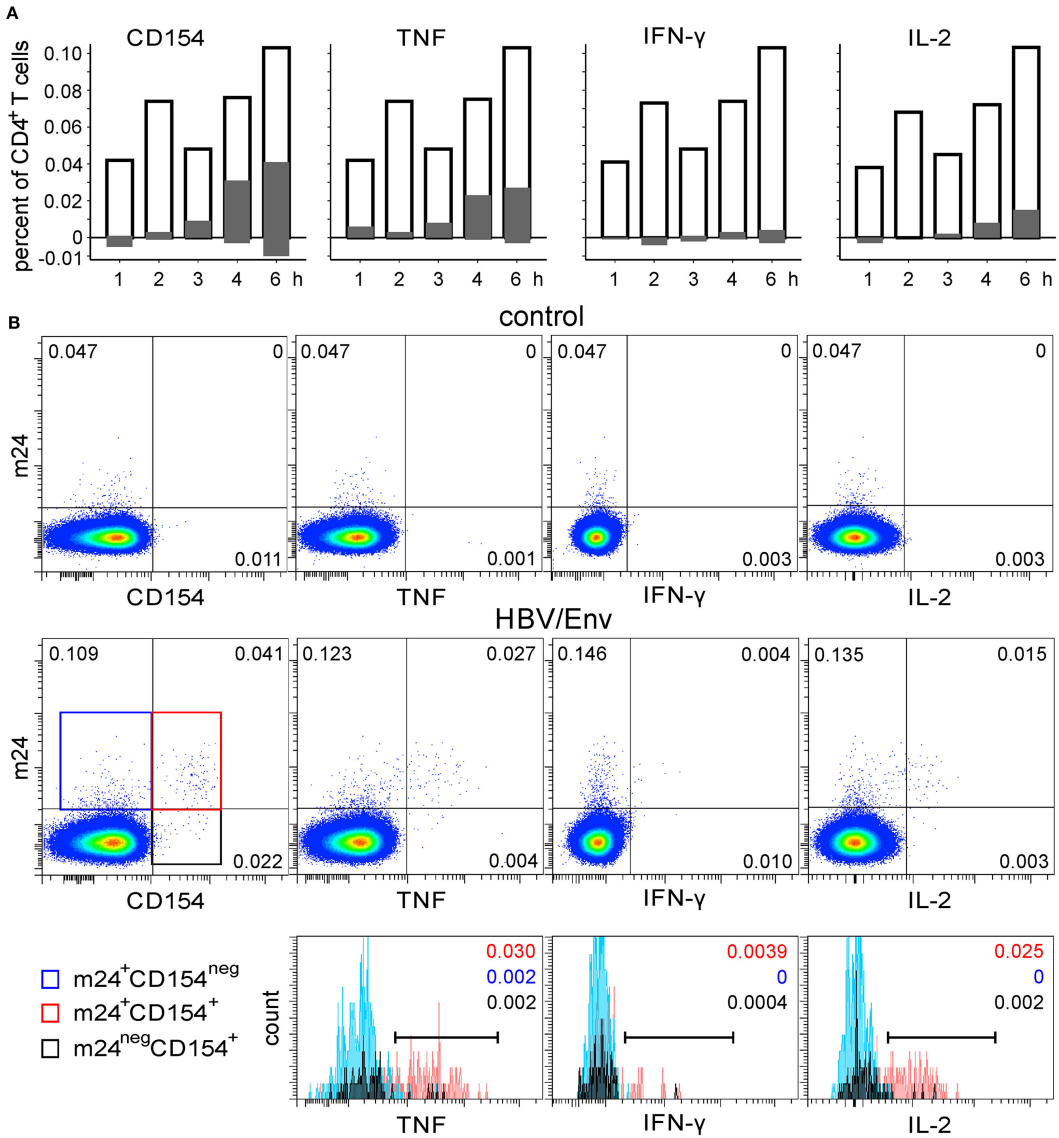
The functional profile of HBV-specific m24^+^ CD4^+^ WB T cells. The WB from a donor who was vaccinated ~10 years ago against HBV was stimulated with HBV/Env overlapping peptides. **(A)** Frequencies of m24 Ab staining, CD154, and cytokine expression within CD4^+^ T cells are shown as bar graphs (at the indicated times and after subtraction of the background assessed in the control test); empty bars represent total m24^+^ cells, positive gray bars represent marker^+^ (CD154, TNF, IFN-γ, or IL-2) m24^+^ CD4^+^ T cells, whereas negative gray bars are marker^+^m24^neg^ CD4^+^ T cells. **(B)** Staining obtained after 6 h without stimulation (control, top) or in the presence of the HBV/Env overlapping peptides (middle). Numbers on density plots indicate frequencies among CD4^+^ T cells. m24^+^ CD154^neg^, m24^+^ CD154^+^, and m24^neg^ CD154^+^ subsets were gated (middle panel: blue, red, and black frames, respectively) and further displayed as histograms according to their TNF, IFN-γ, and IL-2 expression (bottom). Numbers on histograms indicate frequencies of the respective color-coded population among CD4^+^ T cells (markers are shown). Gating strategy is as shown in Figure 1A.

To address specificities against further viruses, the WB from two HBV-vaccinated donors was stimulated with HBV/Env overlapping peptides, was stimulated with SEB (one donor only), or remained unstimulated. The cells were harvested after 1, 2, 3, 4, or 6 h, followed by m24 Ab staining, EDTA treatment, and staining for intracellular CD154 and cytokines upregulation. For the first donor, the time course of HBV/Env stimulation is shown in Figure 6A, and density plots after 6 h of incubation without or with HBV peptides are displayed in Figure 6B top and middle, respectively. Frequencies of HBV-specific cells were low (~0.1% within the CD4^+^ T cell subset), but readily detectable by m24 Ab staining. Similar to what we observed for CMV-specific cells, the CD154 expression and cytokine production were essentially confined to the m24^+^ cell fraction [positive (marker^+^ m24^+^ cells) vs. negative (marker^+^ m24^neg^ cells), Figure 6A, gray bars)]. However, a significant proportion of m24^+^ cells (about 50%) did not upregulate CD154 or cytokines even after 6 h. Some m24^+^ cells negative for the functional markers were also present in the unstimulated control. When we analyzed single and double m24^+^ and CD154^+^ cells, we found that cytokine-producing cells were mainly m24^+^ CD154^+^. Only 6%, 0%, and 0% of the TNF-, IFN-γ-, or IL-2-producing CD4^+^ T cells, respectively, were CD154^neg^, whereas 6%, 9%, and 7% were m24^neg^ (Figure 6B bottom). In unstimulated cells, some cells were m24^+^ or CD154^+^, but no double-stained m24^+^ CD154^+^ events were observed (Figure 6B top, left). After SEB stimulation, a strong association between m24 Ab staining and TNF, IFN-γ, and IL-2 production was observed. Again, a significant proportion of the CD154^+^ cells was m24^neg^; however, those were predominantly cytokine^neg^ (Supplementary Figures 3C,D).

Similar observations were made for a second HBV vaccinee (Supplementary Figure 4). Although some background staining with m24 or CD154 Abs was observed in the unstimulated test, the combination of both markers (0.001% of m24^+^ CD154^+^ cells) enabled distinct detection of HBV-specific cells even at very low frequencies (Supplementary Figure 4B: 0.011, 0.008, 0.001, and 0.004% for CD154, TNF, IFN-γ, and IL-2 dot plots, respectively).

In conclusion, m24 Ab staining is suitable for detecting functional antigen-specific CD4^+^ T cells, and the combination of this marker with the measurement of CD154 upregulation can be used for the identification of extremely low frequencies of functional antigen-specific T cells.

### Monitoring of SARS-CoV-2-Specific T Cell Immunity

**Figure 7:**
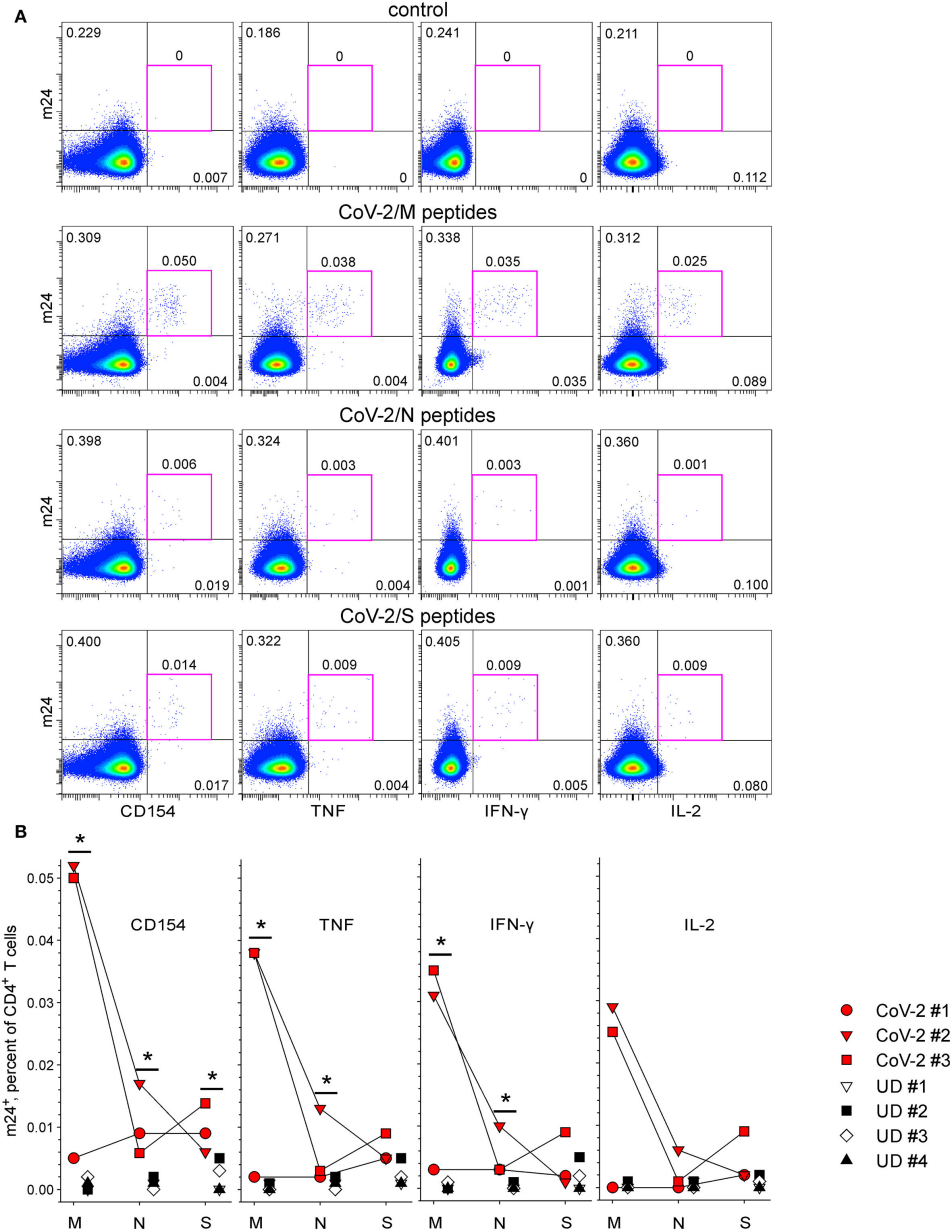
The functional profile of severe acute respiratory syndrome coronavirus 2 (SARS-CoV-2)-specific m24^+^ CD4^+^ WB T cells. m24 Ab staining in combination with CD154, TNF, IFN-γ, or IL-2 expression after stimulation with pools of CoV-2 membrane (M)-, nucleocapsid (N)-, and spike (S)-derived overlapping peptides for 4 h. **(A)** Examples of m24 Ab staining obtained from CoV-2 #3 without stimulation (control) or in the presence of the M, N, or S peptides. Numbers indicate frequencies among CD4^+^ T cells in %. **(B)** The results for marker^+^ m24^+^ CD4^+^ T cells obtained from SARS-CoV-2 convalescents (*n* = 3, CoV-2 #1 to #3) and unexposed healthy controls (*n* = 4, UD #1 to #4) are shown as graphs (after subtraction of background assessed in the control test). Asymptotic significance was calculated using two-tailed Mann–Whitney *U*-tests, *p < 0.05. Numbers indicate frequencies among CD4^+^ T cells in %. Gating strategy is as shown in Figure 1A.

Later, we examined the feasibility of our assay to monitor SARS-CoV-2-specific CD4^+^ and CD8^+^ T cells. Blood was obtained from CoV-2 convalescents (*n* = 3) and unexposed healthy donors (*n* = 4), as confirmed by Ab ELISA on the day of blood withdrawal. In accordance with our previous results for CMV- and HBV-specific CD4^+^ T cells, there were no m24^+^ cells producing CD154 or cytokines without peptide-specific stimulation (0–0.001%, exemplary donor CoV-2 #3 is shown in Figure 7A top, pink frames). The addition of overlapping peptides derived from the membrane (M), N, or spike (S) proteins induced a clear CD4^+^ T cell response in previously exposed individuals, detectable by costaining with m24 and CD154 Ab, even at very low frequencies (down to 0.005–0.006%; M-, N-, and S-specific CD4^+^ T cell responses from donor CoV-2 #3 occurring at frequencies of 0.050, 0.006, and 0.014% are shown in Figure 7A, pink frames). All three individuals responded to the three proteins, albeit at a different intensity. The strongest responses were directed at the protein M (up to ~0.05% of the CD4^+^ T cells in CoV-2 #2 and #3, Figure 7B). CoV-2 #1 responded weakly to protein M (0.005%) but strongly to proteins N and S (both 0.009%). The cytokine production (TNF, IFN-γ, and IL-2) was observed within m24^+^ cells. The T cell activation was virtually not detected in the four unexposed donors (0–0.003%), except for one of them (UD #2) who interestingly showed a distinct, but very low (possibly cross-reactive) response to the protein S alone (0.005% m24^+^ cells expressing either CD154, TNF, or IFN-γ, Figure 7B). Altogether, T cell reactivities against all three proteins tested were significantly increased in the convalescent vs. the non-exposed donor group when considering m24 and CD154 Ab staining together.

**Figure 8:**
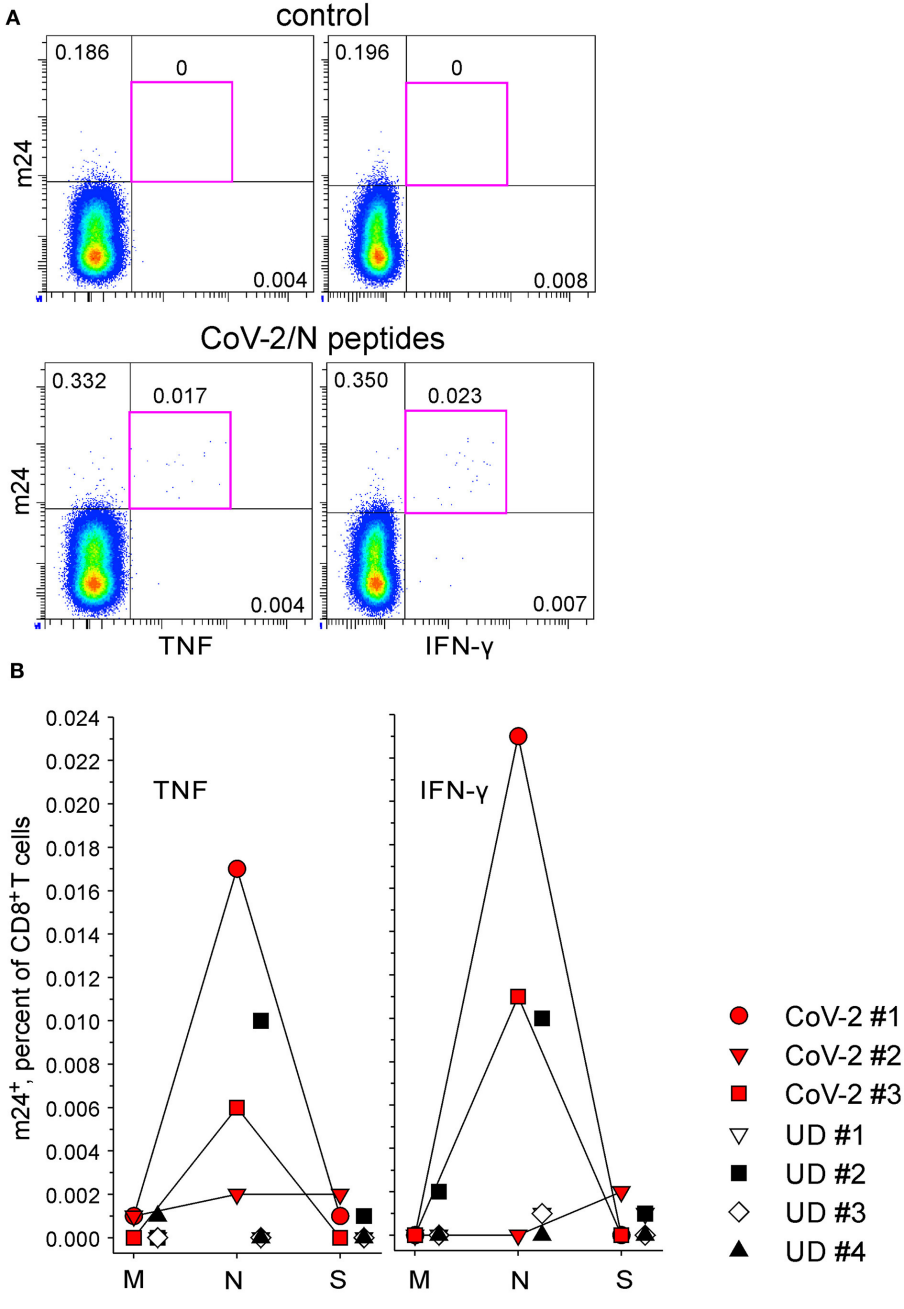
The functional profile of SARS-CoV-2-specific m24^+^ CD8^+^ WB T cells. m24 Ab staining in combination with TNF or IFN-γ expression after 4 h of stimulation with pools of SARS-CoV-2 M-, N-, and S-derived overlapping peptides. **(A)** Examples of m24 Ab staining from CoV-2 #1 obtained without stimulation (control, top) or in the presence of N peptides (bottom). Numbers indicate frequencies among CD8^+^ T cells in %. **(B)** The results for marker^+^ m24^+^ CD8^+^ T cells obtained from SARS-CoV-2 convalescents (*n* = 3, CoV-2 #1 to #3) and unexposed healthy donors (*n* = 4, UD #1 to #4) are shown as graphs (after subtraction of background assessed in the control test). No significant difference was found using two-tailed Mann–Whitney *U*-tests. Numbers indicate frequencies among CD8^+^ T cells in %. Gating strategy is as shown in Figure 2A.

The anti-SARS-CoV-2 CD8^+^ T cell reactivity was assessed simultaneously to the CD4^+^ T cell measurement (m24 Ab staining after 4 h of stimulation). There were no m24^+^ cells producing cytokines without antigen-specific stimulation (0–0.001%, shown for CD8^+^ T cells from CoV-2 #1 in Figure 8A, top). Interestingly, only protein N was recognized in two out of three convalescents, and m24^+^ cells produced TNF (0.017 and 0.006% for CoV-2 #1 and CoV-2 #3, respectively) and IFN-γ (0.023 and 0.011%) (Figure 8B) but not IL-2 (≤ 0.001%). None of the four unexposed volunteers reacted to any of the CoV-2 peptides (0–0.002%), except again for UD #2 who had a response to the N-derived peptide pool (0.010% m24^+^ CD8^+^ T cells expressing either TNF or IFN-γ).

**Figure 9:**
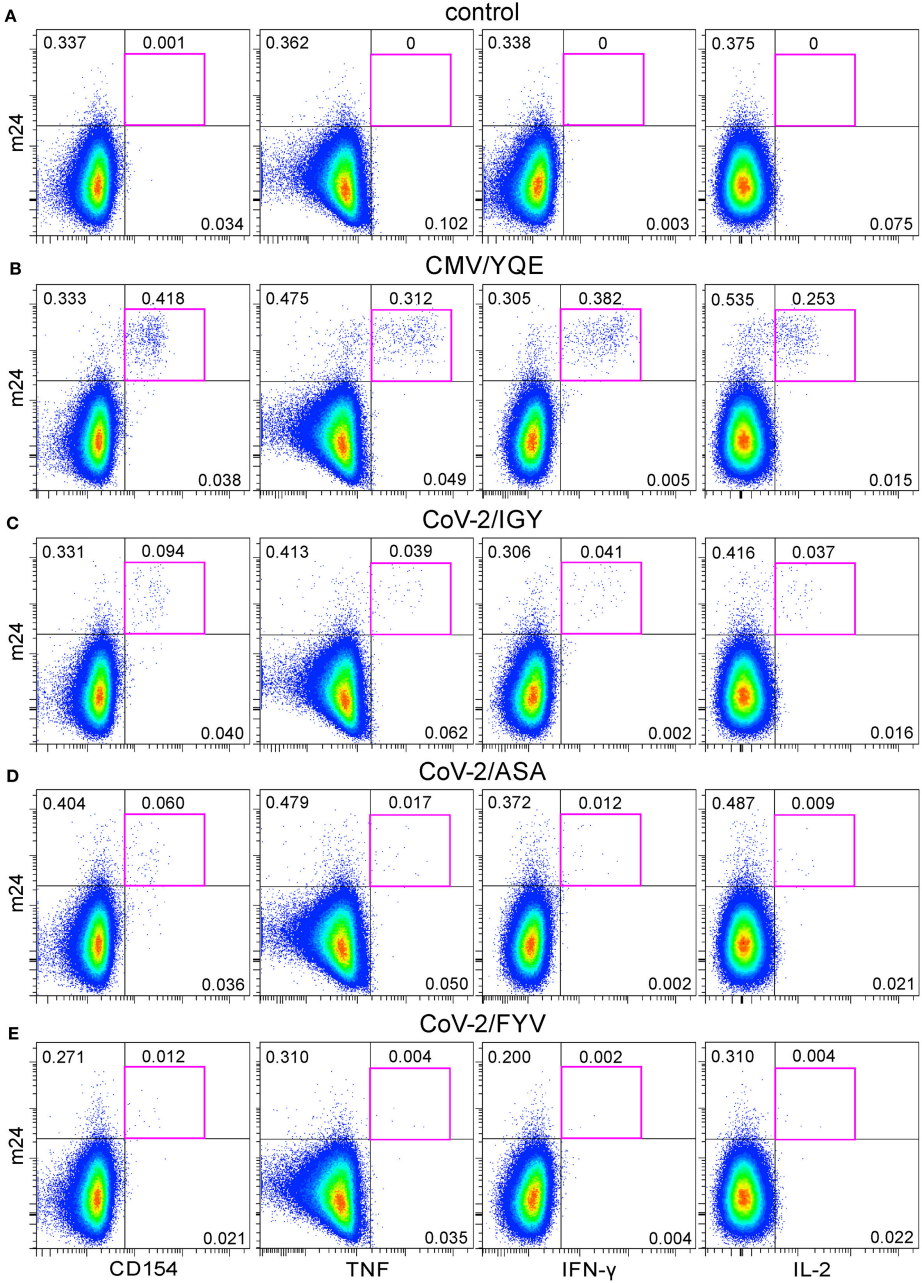
The functional profile of CMV- and SARS-CoV-2-specific CD4^+^ WB T cells after peptide vaccination. **(A–E)** m24 Ab staining in combination with CD154, TNF, IFN-γ, or IL-2 expression obtained from a donor vaccinated with one CMV and three SARS-CoV-2 major histocompatibility complex (MHC)-class II peptides without stimulation (control, **A**), with CMV/YQE **(B)**, CoV-2/IGY **(C)**, CoV-2/ASA **(D)**, or CoV-2/FYV peptides **(E)** for 6 h stimulation. Numbers indicate frequencies among CD4^+^ T cells in %. Gating strategy is as shown in Figure 1A.

We next tested CD4^+^ T cell reactivity in the blood of a healthy volunteer who had been immunized ~6 weeks before with a cocktail of synthetic peptides containing HLA-class II SARS-CoV-2 sequences derived from the N protein (CoV-2/IGY and CoV-2/ASA) and from the Env protein (CoV-2/FYV), as well as one recall CMV pp65 peptide (CMV/YQE) [(13) Preprint]. Virtually, no m24^+^ cells producing CD154 or cytokines were detected when cells were left unstimulated for 6 h (0–0.001%, Figure 9A). The CMV/YQE stimulation caused a strong expression of CD154 (0.418%), TNF (0.312%), IFN-γ (0.382%), and IL-2 (0.253%) within m24^+^ cells (Figure 9B). The three SARS-CoV-2 peptides used for vaccination induced variable responses, with 0.094, 0.060, and 0.012% of m24^+^ CD154^+^ CD4^+^ T cells for SARS-CoV-2 N/IGY, N/ASA, and Env/FYV, respectively, as well as for the production of all three cytokines (Figures 9C–E). These results are coherent with those obtained by IFN-γ ELISpot ~3 weeks after immunization [(13) Preprint].

### m24 Ab Staining of Antigen-Specific CD4^+^ and CD8^+^ T Cells in Frozen/Thawed PBMCs

We have also tested our method for the detection of T cells within cryopreserved PBMC samples. First, two donors, with high (~2% of DRB1^*^11/HPT tetramer^+^) and low (~0.05% CD154^+^ against HBV/Env) frequencies of virus-specific CD4^+^ T cells, were selected (experiments with WB of the same donors are reported in Figures 3, 5, 6). PBMCs were stimulated with the CMV/HPT peptide (first donor), were stimulated with HBV/Env overlapping peptides (second donor), or remained unstimulated for 6 h, followed by incubation with m24 Ab plus EDTA treatment and staining for intracellular CD154 and cytokines (TNF, IFN-γ, and IL-2).

For the first donor, β_2_-integrin activation (m24 Ab binding) and functionality (CD154, TNF, IFN-γ, and IL-2 production) were stronger in PBMCs than in WB (1.90 vs. 1.36%, respectively, of m24^+^ CD154^+^ cells; compare Supplementary Figure 5B and Figure 5B). Similar to what we observed with WB (Figure 5A), the upregulation of CD154 and cytokines was exclusively confined to the m24^+^ cell subset and almost all m24^+^ cells (93%) expressed CD154, TNF, or IFN-γ (Supplementary Figure 5B). Moreover, cytokine-producing cells were mainly m24^+^ CD154^+^. Only 17, 12, and 1% of the TNF-, IFN-γ-, or IL-2-producing cells did not express CD154, and virtually none were m24^neg^ (Supplementary Figure 5B bottom). Similar observations were made for the second donor, with a more robust response in PBMCs than in WB (0.058 vs. 0.041% of m24^+^ CD154^+^ cells for PBMCs and WB, respectively; compare Supplementary Figure 5C and Figure 6B). Again, some single-stained m24^+^ or CD154^+^ events were observed in unstimulated cells, but virtually no double-stained m24^+^ CD154^+^ events were detected (Supplementary Figure 5C top, left). Almost none of the TNF-, IFN-γ-, or IL-2-producing CD4^+^ T cells were CD154^neg^, whereas only 1, 0, and 8% were m24^neg^, respectively (Supplementary Figure 5C bottom). Thus, m24 Ab staining together with the expression of CD154 allows the reliable identification of low frequency, polyfunctional CD4^+^ T cells in cryopreserved PBMCs.

**Figure 10:**
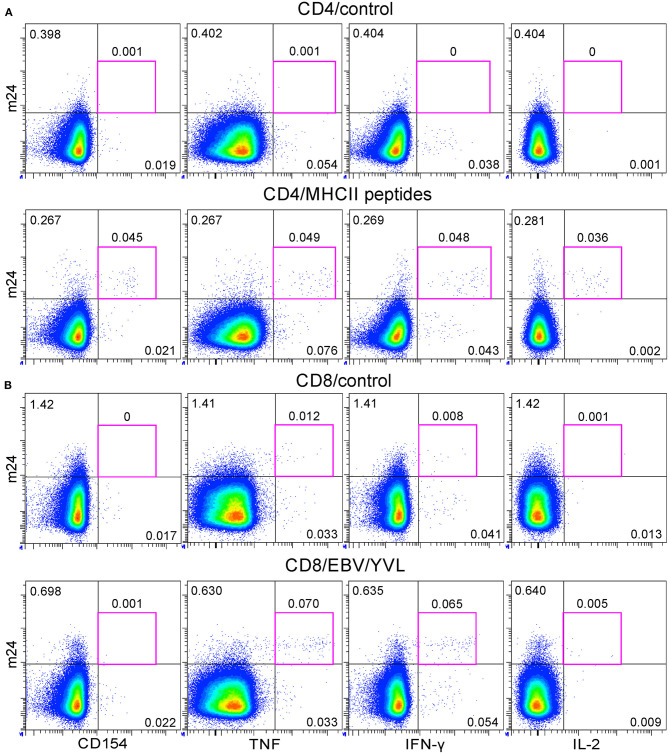
The application of the m24 Ab assay for simultaneous detection of antigen-specific CD4^+^ and CD8^+^ T cells within cryopreserved peripheral blood mononuclear cells (PBMCs). PBMCs of a preselected healthy donor were thawed and rested overnight before a 4 h stimulation with a pool of HLA-class II and HLA-A*02 peptides. **(A)** m24 Ab staining and CD154, TNF, IFN-γ, and IL-2 expressions for unstimulated (control) or major histocompatibility complex (MHC)-class II peptide-stimulated CD4^+^ T cells (mix of *n* = 9 virus-derived peptides). **(B)** The same stainings for CD8^+^ T cells without stimulation (control) or after stimulation with a HLA-A*02 epitope (YVLDHLIVV and EBV/YVL). Gating strategy is similar to that of Figures 1A, 2A, with Zombie Aqua included as a live/dead cell marker. See also Supplementary Figure 5A.

We finally applied the m24 Ab assay for simultaneous detection of antigen-specific CD4^+^ and CD8^+^ T cells within PBMCs of one prescreened donor (with a known low CD4^+^ T-cell reactivity against an HLA-class II peptide mix containing CMV-, EBV-, and Flu-derived epitopes, and a low frequency of CD8^+^ T cells against one HLA-A^*^02 restricted EBV-derived epitope). Background staining was observed with single m24 or single CD154 Abs on unstimulated CD4^+^ T cells; however, the combination of both markers allowed to readily detect ~0.05% of reactive CD4^+^ T cells (Figure 10A), whereas the combination of m24 Ab with TNF and IFN-γ (but not with CD154 or IL-2) sensitively identified EBV-specific CD8^+^ T cells (Figure 10B).

## Discussion

**Figure 11:**
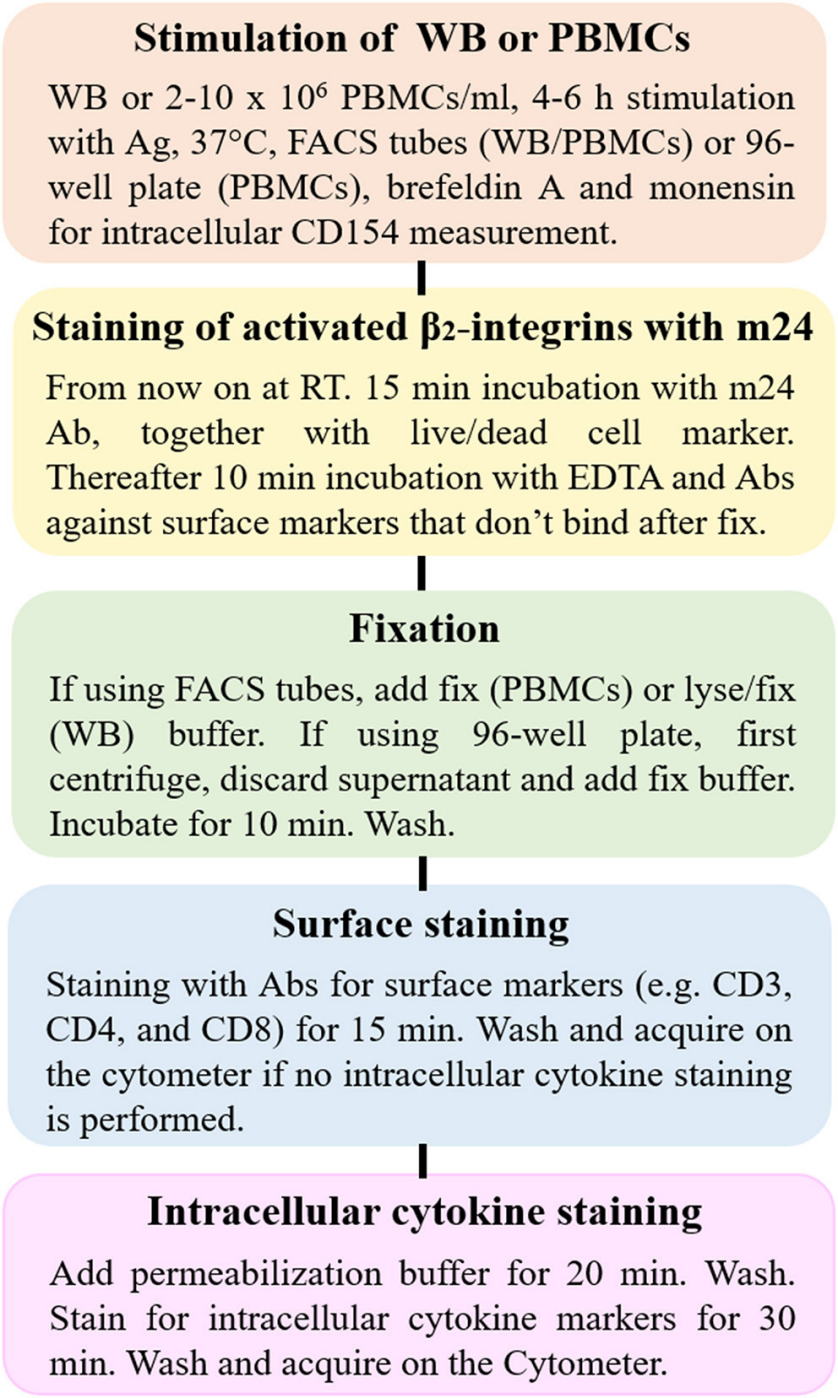
The scheme of experimental procedure for flow cytometry-based detection of activated CD4^+^ and CD8^+^ T cells using m24 Ab. The procedure can be performed on WB or PBMCs, either in fluorescence-activated cell sorting (FACS) tubes (WB and PBMCs) or 96-well plates (PBMCs) and is divided into five main steps: (1) antigen-specific stimulation, (2) staining of activated β_2_-integrins with m24 Ab, followed by EDTA treatment, (3) fixation/erythrocyte lysis of WB or fixation of PBMCs, (4) cell surface marker staining, and (5) permeabilization and intracellular staining. Additional modifications can be made for covering different specific questions as indicated in the scheme.

We describe a protocol for sensitive assessment of virus-specific CD4^+^ T cells, which, comparable to our prior ICAM-1 multimer assay for flow cytometry-based detection of CD8^+^ T cells, takes advantage of the conformational change of membrane-bound β_2_-integrins after the activation. Instead of multimers of the integrin ligands, the modified method uses a monoclonal Ab (m24 Ab) specific for the open, high-affinity conformation of activated β_2_-integrins. The m24 Ab detects very low numbers of effector CD4^+^ and CD8^+^ T cells in WB or cryopreserved PBMCs specific for a range of viruses (i.e., SARS-CoV-2, CMV, EBV, and HBV). The combination of m24 and CD154 Ab gives a remarkable low background in unstimulated samples (≤ 0.002% of CD4^+^ T cells), allowing the reliable detection of antigen-specific CD4^+^ T cells at extremely low frequencies (down to 0.004%). This is far below the lower detection limit of the standard flow cytometry assays and in the range of what can be resolved with combinatorial approaches (6, 17–19). A scheme summary of our suggested experimental procedure for detection of activated CD4^+^ and CD8^+^ T cells using m24 Ab is given in Figure 11.

While the activation of β_2_-integrins on CD8^+^ T cells occurs within several minutes of peptide stimulation (5), we show that several hours are needed for CD4^+^ T cells. This difference might reflect specificities in the integrin activation pathways of the two main T lymphocyte subsets (e.g., differences in the expression of adaptor molecules downstream of the TCR signaling pathway). It might also explain their distinct patterns of immunological synapse formation (transient vs. stable), which may be adapted due to their ultimate main functions (serial killing of numerous target cells vs. sustained cytokine production) (10). We know that rapid integrin activation can be found on various differentiation stages of memory and effector CD8^+^ T cells (5). Preliminary experiments for CD4^+^ T cells using CD27, CD28, and CCR7 markers indicate that activated β_2_-integrins after 4–6 h of stimulation mark all CD4^+^ T cells irrespective of their differentiation stages (data not shown). However, it cannot be excluded that certain subsets require an even longer time for the activation of β_2_-integrins.

Although several hours of stimulation are needed for the β_2_-integrin activation on CD4^+^ T cells, this event is still detectable earlier than the conventional flow cytometry markers on CD4^+^ T cells, such as CD154 and cytokines. We observed that functional (i.e., cytokine producing) CD4^+^ T cells were contained exclusively within the m24^+^ fraction, demonstrating that robust activation of β_2_-integrins reveals highly effective, functional antigen-specific CD4^+^ T cells. The intensity of m24 Ab staining on CD4^+^ T cells is relatively dim and has some background staining. However, the combination with other markers, for example, CD154, increases the signal-to-noise ratio, allowing detection of very low frequencies of CD4^+^ antigen-specific T cells. m24 Ab stained surface activated integrins, whereas CD154 was determined intracellularly together with a range of cytokines after stimulation in the presence of brefeldin A and monensin as previously described in Frentsch et al. (8). To simplify the assay, an option might be the cell surface staining of both activated β_2_-integrins and CD154, provided that a CD40-specific Ab is added to the culture to stabilize extracellular CD154 neo-expression (8).

EDTA treatment was required to visualize singlet responding CD4^+^ T cells, suggesting intense cell clustering between T helper and HLA-class II^+^ antigen-presenting cells. Since EDTA reversed the binding of ICAM-1 multimers to the activated β_2_-integrins, we used the m24 Ab whose binding was not affected by subsequent EDTA treatment. Similar to the detection with ICAM-1 multimers (5), staining of the β_2_-integrin activation with m24 Ab on CD8^+^ T cells was maximal after 4–16 min of stimulation with peptides. However, in contrast to the ICAM-1 multimers, m24 Ab binding on antigen-specific CD8^+^ T cells diminished only slightly following prolonged activation (4–6 h). Therefore, m24 Ab staining can be used for simultaneous screening of both CD4^+^ and CD8^+^ T cell reactivities. Since the fluorescence intensity of m24 Ab is generally dimmer than that of ICAM-1 multimers, we however recommend to perform staining after a short-term stimulation with ICAM-1 multimers (without EDTA treatment) when only antigen-specific CD8^+^ T cells are assessed [see Figure 2 and Supplementary Figure 10 from Schöllhorn et al. [(20) Preprint]].

To use the assay for clinical samples, it was important to optimize the protocol for the detection of T cells not only in WB but also in cryopreserved PBMCs. We used 2 × 10^6^ PBMCs in 1 ml medium per test in FACS tubes, which was suitable for the detection of antigen-specific CD4^+^ and CD8^+^ T cells after a 4–6 h stimulation. If needed, upscaling of the cell concentration is possible without major problems. This is in contrast to the short-term stimulation protocol for the detection of antigen-specific CD8^+^ T cells alone. In the short term stimulation, we observed that the PBMC concentration (2 × 10^6^ cells in 1 ml or 0.2 ml medium) influenced detection due to heavy aggregation in the presence of activating peptide when the higher PBMC concentration was used [see Supplementary Figures 6–9 from Schöllhorn et al. [(20) Preprint]]. Another observation we made was that frequencies of antigen-specific CD4^+^ T cells were higher within PBMCs than when using WB, possibly reflecting different cell–cell interaction conditions in the two cell sources. We also observed that the method is transferable to 96-well plates, which allows easier handling of many tests simultaneously. By repeated testing of the very same donor and specificity (~0.2% of the CD4^+^ subset), we also observed excellent intra- (triplicate tests) and inter-experiment (repetition on 3 days) variability, with percentage coefficients of variation ranging from ~3 to 13%, which is in the range of other functional T cell assays (data not shown).

As a currently highly relevant research topic, we evaluated CD4^+^ and CD8^+^ antigen-specific T cells in SARS-CoV-2 convalescents in comparison to unexposed healthy donors. CD4^+^ T cells specific for S, N, and M proteins were detected in all three convalescents, whereas CD8^+^ T cells were detected only toward the N protein. In line with other recent reports, we also observed in one unexposed donor very low frequencies of CD4^+^ and CD8^+^ T cells against S and N proteins, respectively, probably due to cross-reactive cells primed against other SARS viruses (21–23). We also used m24 Ab staining to confirm the induction of anti-vaccine CD4^+^ T cells after experimental immunization of one healthy volunteer with synthetic peptides from the S and N proteins [(13) Preprint]. Importantly, the results matched those of a previous IFN-γ ELISpot, and our method allowed reliable detection of the weakest anti-peptide response (0.012% m24^+^ CD154^+^ CD4^+^ T cells vs. 29 spots/300,000 PBMCs against CoV-2/FYV peptide).

**Figure 12:**
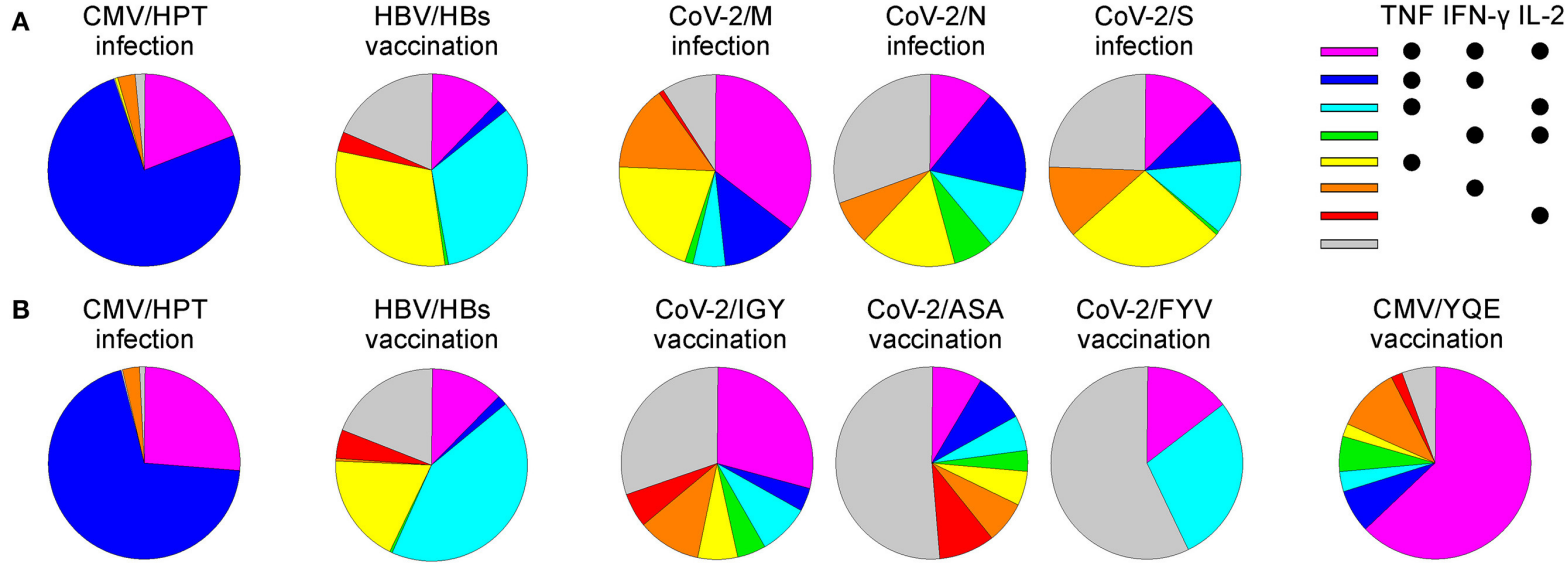
The polyfunctional profile of CD154^+^ m24^+^ CD4^+^ WB T cells for various viruses and vaccination conditions. The pie charts represent the distribution of the cytokine production patterns from the donors and antigens tested in the study after CMV peptide vaccination (*n* = 1), CMV infection (*n* = 1), CoV-2 infection (*n* = 3, mean is represented), CoV-2 peptide vaccination (*n* = 1), or HBV vaccination (*n* = 2, mean is represented) after 4 h **(A)** or 6 h **(B)** of stimulation with the respective peptides. Samples of the CMV infection and HBV vaccination were stimulated for 4 and 6 h for comparative purposes.

The m24 Ab identifies functional cells (i.e., those that produce cytokines upon stimulation). Interestingly, different CD4^+^ antigen specificities showed unique cytokine profiles, which were also distinct in post infection vs. vaccination conditions (Figure 12). Chronically CMV-infected individuals showed a prevalent TNF and IFN-γ production pattern, with some IL-2, while CMV-peptide boost vaccination was predominated by a triple cytokine production with higher IL-2 levels. The HBV vaccination selected for a TNF-dominant memory cell pool that was almost equally distributed in the TNF production alone, together with IL-2, or with both IL-2 and IFN-γ. SARS-CoV-2 convalescents had a more heterogeneous functional profile of either single, double, or triple cytokine-producing cells, possibly reflecting a peculiar immune response to the virus and/or recent infection. Only the response to the M protein was dominated by triple-positive cells. Finally, the vaccination response to one of the three CoV-2 peptides (CoV-2/IGY) was not only the strongest but also characterized by the highest proportion of triple cytokine positive cells. Thus, *ex vivo* costaining for activated integrins and CD154 on very low frequencies of effector/memory CD4^+^ T cells allows gaining precise information about the polyfunctionality of the immune response. This is of importance since prior findings showed that the magnitude of the vaccine-induced polyfunctionality highly correlates with protection against pathogens (24–26). Although we essentially analyzed Th1 cells, including Abs against further cytokines could be useful to dissect the functional heterogeneity and refine Th profiles of pathogen- or vaccine-specific CD4^+^ T cell responses (27).

In conclusion, we present a cytometry-based assay for the identification of antigen-specific CD4^+^ and CD8^+^ T cells. For CD4^+^ T cells, in particular, costaining of β_2_-integrins with the activation marker CD154 allows the detection of extremely low frequencies of responding polyfunctional cells. The assay can be used for highly sensitive screening of T cell reactivities, for example, in epitope mapping studies, as they are currently performed for the new SARS-CoV-2 virus or in translational approaches.

## Data Availability Statement

The raw data supporting the conclusions of this article will be made available by the authors, without undue reservation.

## Ethics Statement

The studies involving human participants were reviewed and approved by Ethics Committee of the University of Tübingen. The patients/participants provided their written informed consent to participate in this study.

## Author Contributions

AS, JS, LB, TL, H-GR, JB, CG, and SD conceived the study and designed the experiments. AS, RF, CG, and SD conducted the experiments. AS, JS, CG, and SD performed the data analysis, prepared the figures, and wrote the manuscript. AJ and SS provided critical reagents. CG and SD supervised the study. All authors contributed to the article and approved the submitted version.

## Conflict of Interest

The authors declare that the research was conducted in the absence of any commercial or financial relationships that could be construed as a potential conflict of interest.

## Supplementary Material

Supplementary file contains five Figures 1–5.
