# Supplementary material for: Integrin Activation Enables Sensitive Detection of Functional CD4+ and CD8+ T Cells: Application to Characterize SARS-CoV-2 Immunity
Source: Front Immunol. 2021 Mar 29;12:626308. doi: 10.3389/fimmu.2021.626308 (PMC8040333; doi:10.3389/fimmu.2021.626308)
Supplement: Supplementary file 1 [file Data_Sheet_1.docx]

**Supplementary Figures**

**
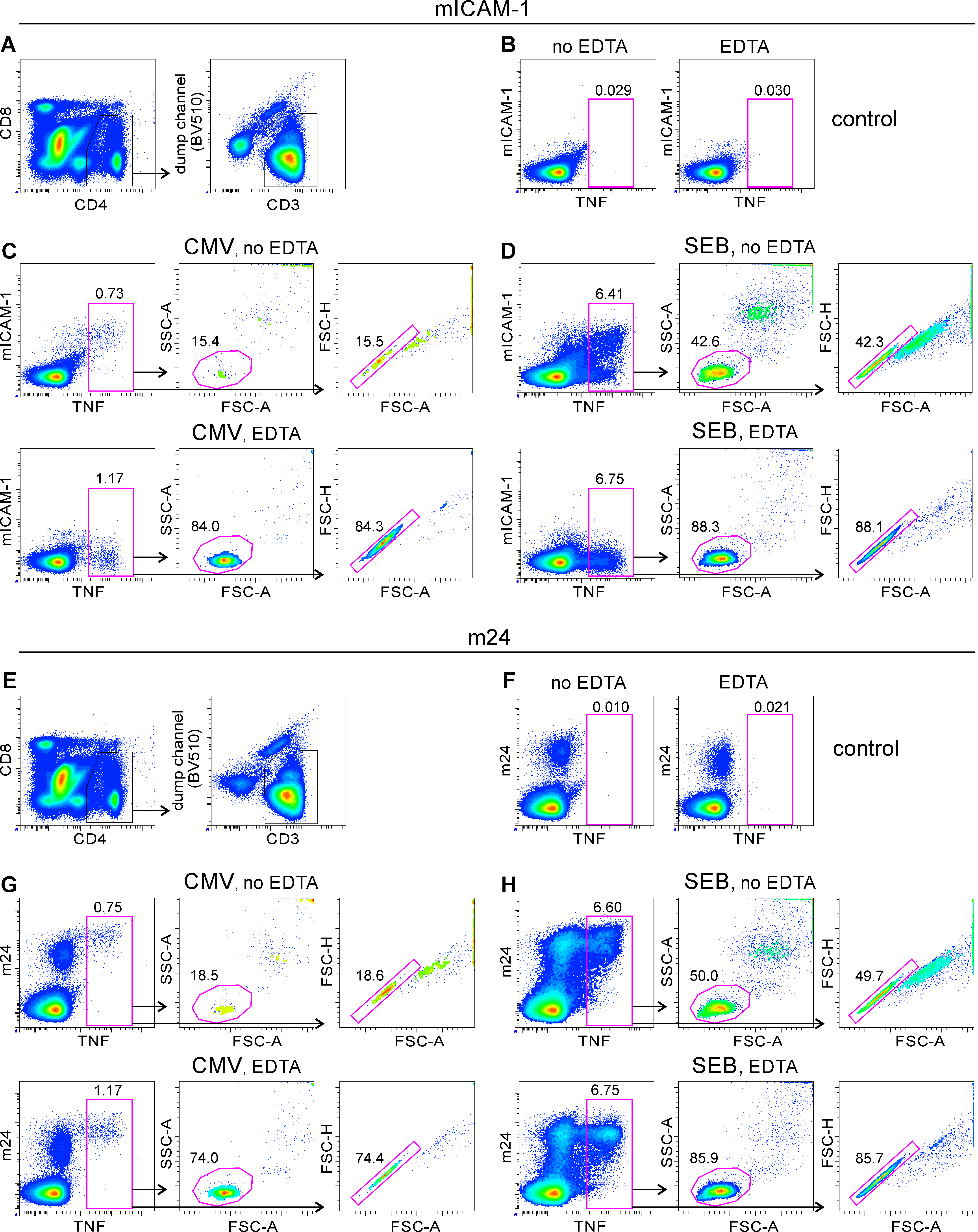
**

**Fig. S1.** Detection of TNF^+^ CD3^+^ CD4^+^ WB T cells within the lymphocyte and singlet gates in relation to EDTA treatment. The figure shows results obtained for mICAM-1/m24 Ab stained samples and analysis of basic parameters FSC and SSC. (A and E) Gating strategy. From left to right, the gating of CD4^+^ events and of CD3^+^ T cells. (B-D and F-H) WB cells were cultured without stimulus (B, F), with the CMV/HPT peptide (C, G) or with SEB (D, H). TNF^+^ cells within the CD4^+^ T cells were defined (left, pink frame, frequencies are indicated) and their distribution on FSC-A/SSC-A plots (middle) or FSC-A/FSC-H plots (right) without (top) or with EDTA treatment (bottom) after the staining with mICAM-1 (B-D) or m24 Ab (F-H) was assessed. Numbers on the FSC-A/SSC-A and FSC-A/FSC-H plots indicate the percentages of TNF^+^ CD4^+^ T cells falling within the gates (pink). Note that results are partially shown in Fig.1, but here the FSC-A/SSC-A lymphocyte gate and the FSC-A/FSC-H duplet exclusion are not included in the gating strategy. Without lymphocyte pregating, a population of m24^+^ TNF^neg^ cells is visible also in the unstimulated control. These are very large events containing probably aggregated T cells and monocytes (the latter binding to m24 Ab after stimulation).


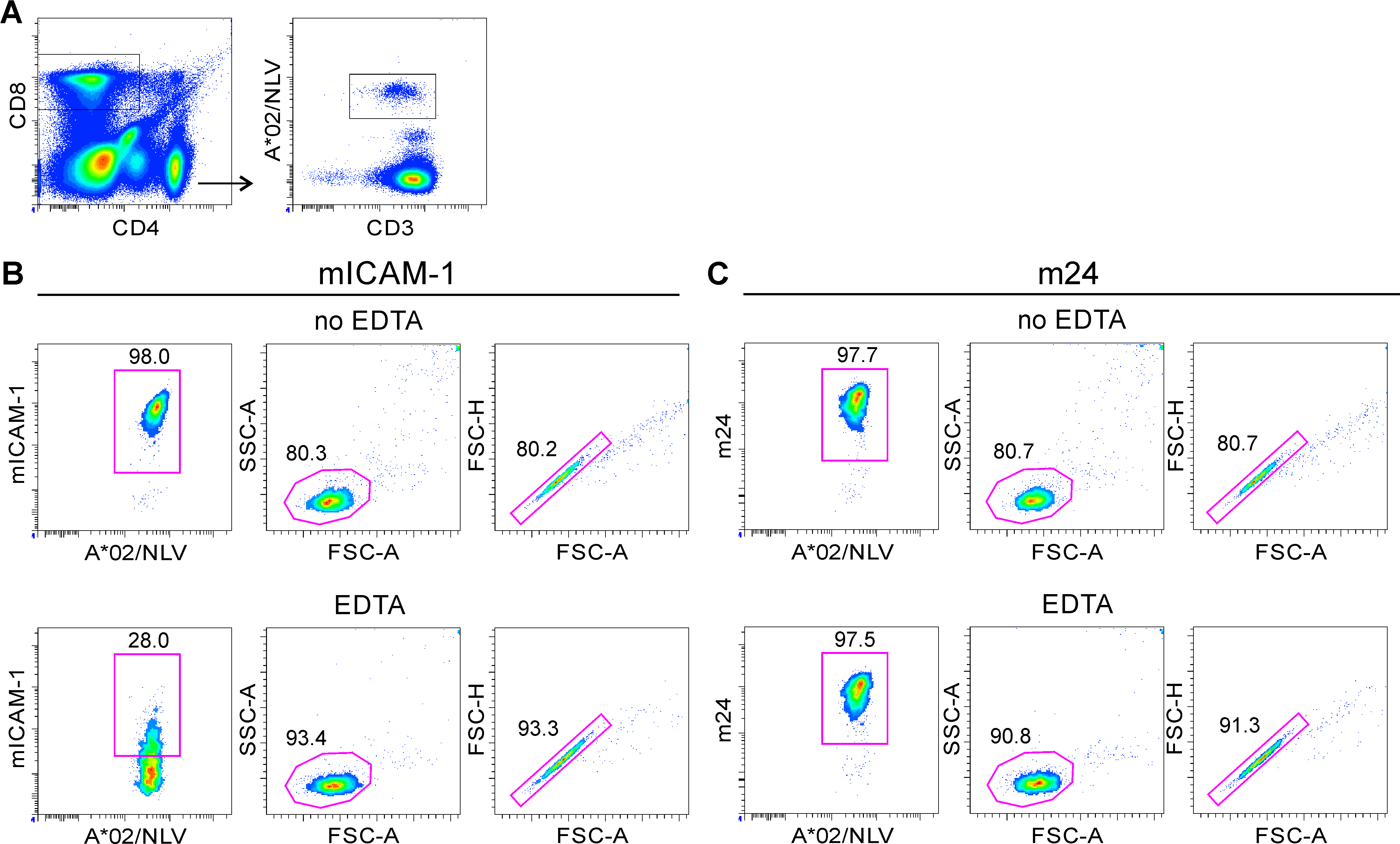


**Fig. S2.** Detection of HLA-A*02 restricted, CMV/NLV-specific CD8^+^ WB T cells (costained with CD3, CD4, CD8 Abs, and mICAM-1 or m24 Ab) within the lymphocyte singlet cell gate does not depend on the EDTA treatment. (A) Gating strategy. From left to right: the gating of CD8^+^ events and tetramer A*02/NLV^+^ CD3^+^ T cells. (B, C) β_2_-integrin activation on A*02/NLV^+^ cells (left) and distribution within FSC-A/SSC-A lymphocyte plots (middle) or FSC-A/FSC-H singlet plots (right), without (top) or with EDTA treatment (bottom) after the staining with mICAM-1 (B) or m24 Ab (C). Numbers indicate the percentages of A*02/NLV^+^ CD8^+^ T cells falling within the gates (pink). Note that results are partially shown in Fig.2, but here the FSC-A/SSC-A lymphocyte gate and the FSC-A/FSC-H duplet exclusion are not included in the gating strategy.


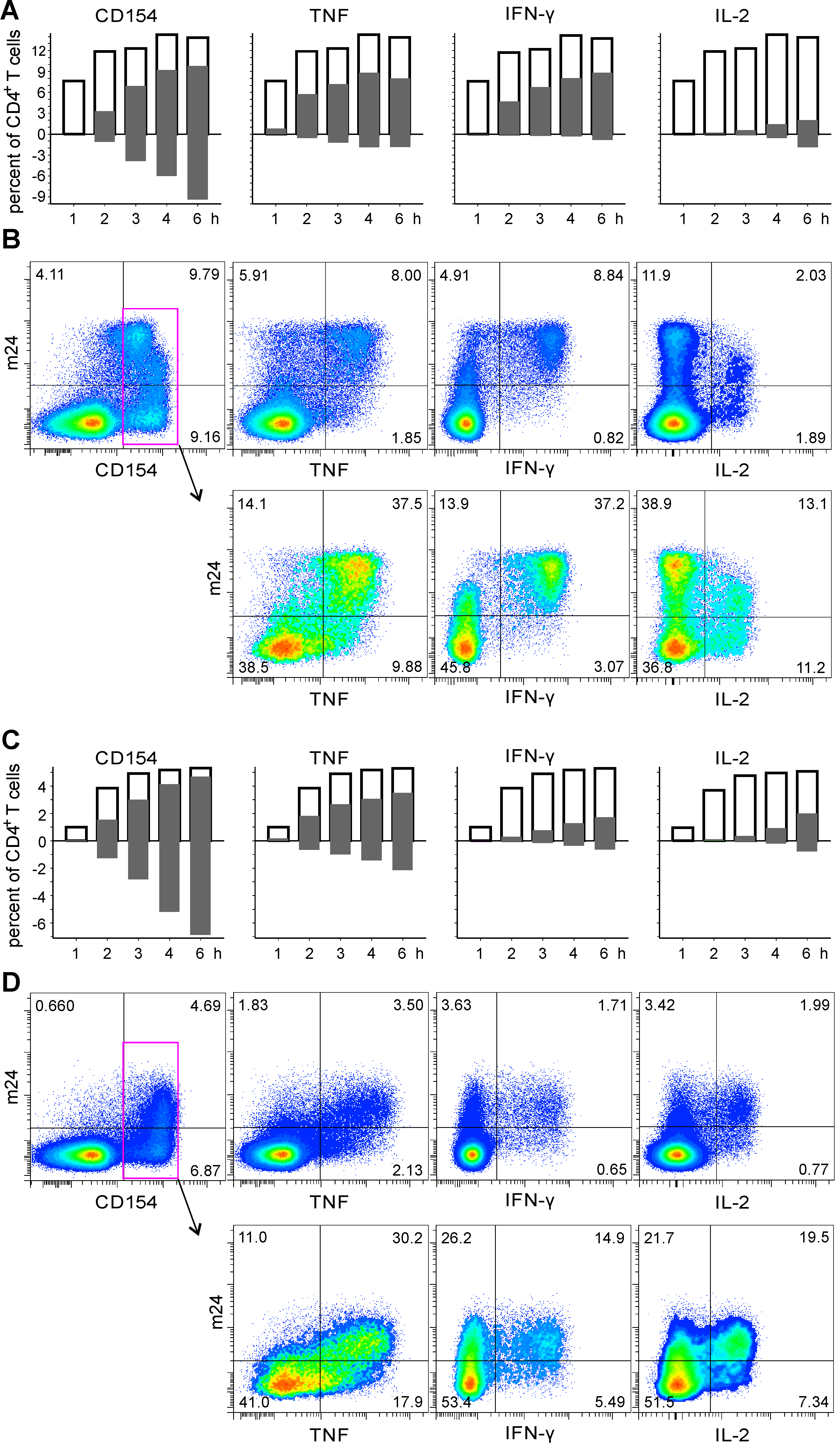


**Fig. S3.** Functional profile of SEB-stimulated m24^+^ CD4^+^ WB T cells. m24 Ab staining for the two donors DRB1*11^+^ and HBV-vaccinee shown in Figs. 5 and 6, respectively. (A, C) The results of the SEB stimulations are shown as graphs (at the indicated times and after subtraction of the background assessed in the control test; frequencies within CD4^+^ T cells are shown); empty bars represent total m24^+^ cells, positive gray bars represent marker^+^ (CD154, TNF, IFN-γ, or IL-2) m24^+^ CD4^+^ T cells, while negative gray bars marker^+^ m24^neg^ CD4^+^ T cells. (B, D) m24 Ab staining after 6 h stimulation (top panels). CD154^+^ m24^neg^ and m24^+^ subsets (pink frame) are further displayed according to TNF, IFN-γ, and IL-2 expression (bottom). Numbers on density plots indicate frequencies among total or CD154^+^ CD4^+^ T cells. Gating strategy is as in Fig. 1*A*, and control stains are shown in Figs. 5 and 6.


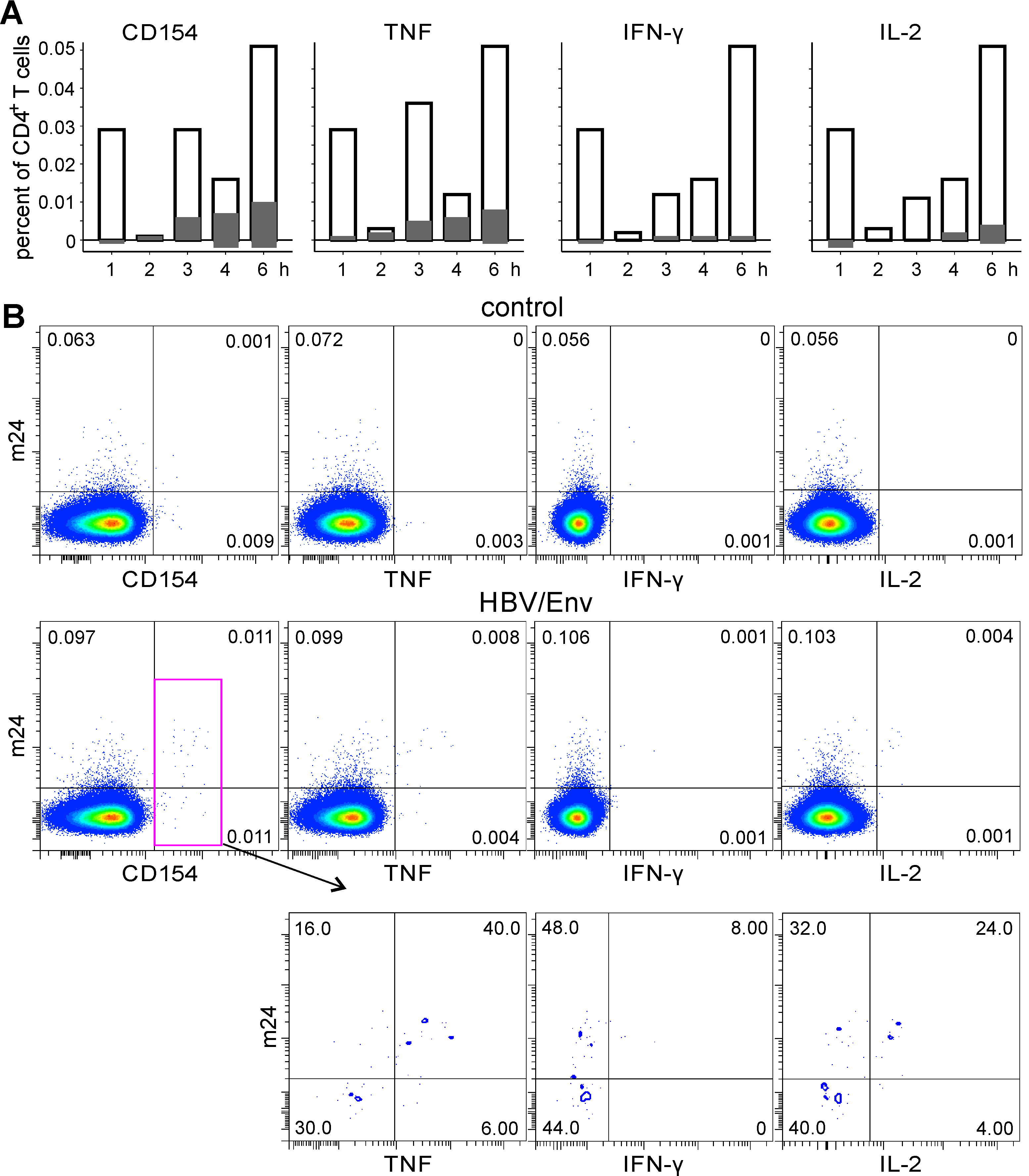


**Fig. S4.** Functional profile of HBV-specific, m24^+^ CD4^+^ WB T cells from a second vaccinee. Cell stimulation was performed with HBV/Env overlapping peptides. (A) Results are shown as graphs (at the indicated times and after subtraction of the background assessed in the control test; frequencies within CD4^+^ T cells are shown); empty bars represent total m24^+^ cells, positive gray bars represent marker^+^ (CD154, TNF, IFN-γ, or IL-2) m24^+^ CD4^+^ T cells, while negative gray bars marker^+^ m24^neg^ CD4^+^ T cells. (B) m24 Ab staining after 6 h without stimulation (top) or in the presence of HBV/Env peptides (middle). CD154^+^ m24^neg^ and m24^+^ subsets (pink frame) are further displayed according to TNF, IFN-γ, and IL-2 expression (bottom). Numbers on the density plots indicate frequencies among total or CD154^+^ CD4^+^ T cells. Gating strategy was as in Fig. 1*A*.


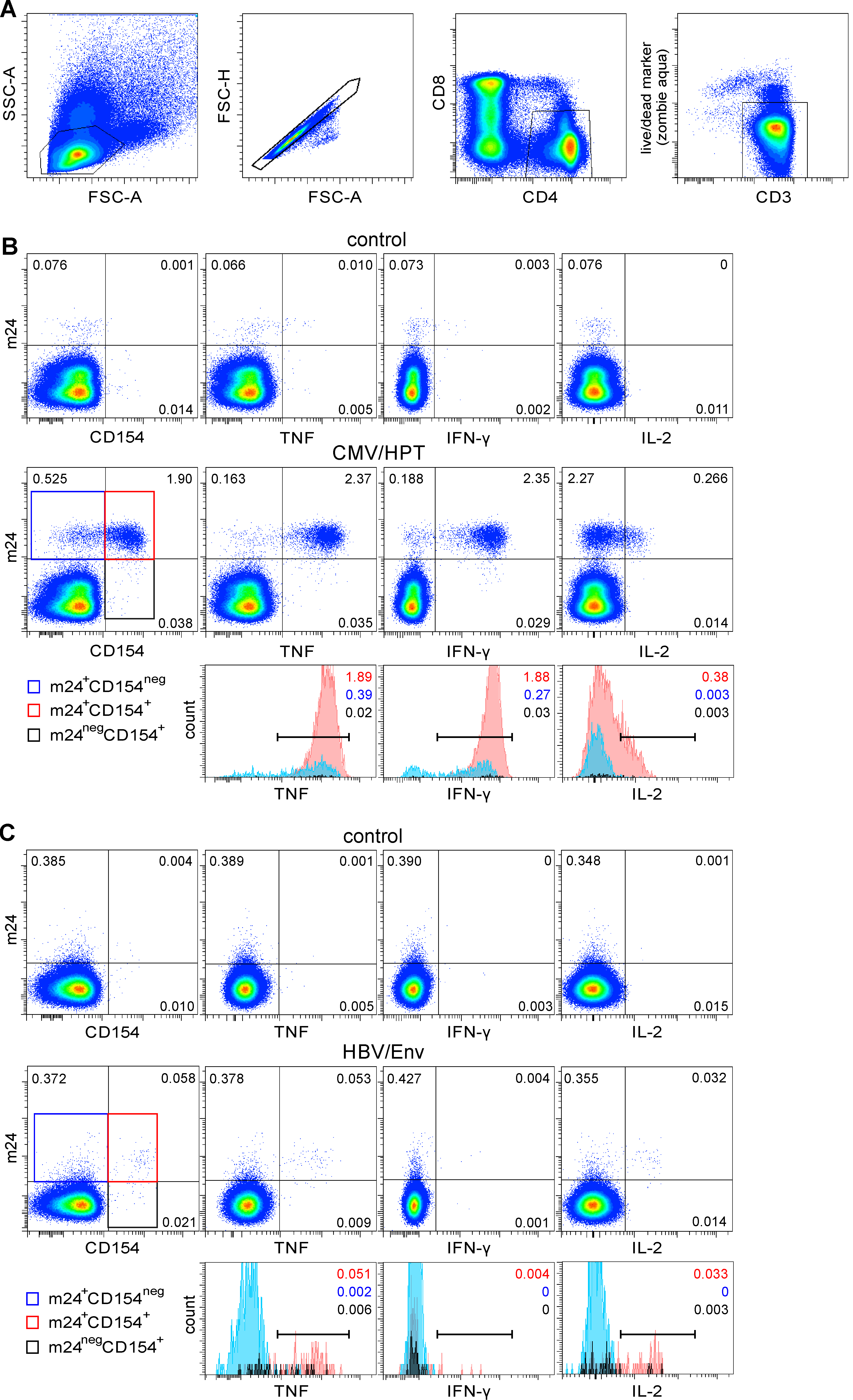


**Fig. S5.** Assessment of CMV- and HBV-specific CD4^+^ T cells in frozen/thawed PBMCs. Donors are the same as in Figs. 5 and 6, respectively. (A) Gating strategy. From left to right, the lymphocyte gate, the FSC-A/H duplet exclusion, the gating of CD4^+^, and of living CD3^+^ T cells. (B, C) m24 Ab staining in combination with CD154, TNF, IFN-γ or IL-2 expression (from left to right) after 6 h stimulation of 2x10^6^ PBMCs (in 1 ml TCM) without (control), with CMV/HPT (B) or with HBV/Env peptide pool (C). m24^+^ CD154^neg^, m24^+^ CD154^+^ and m24^neg^ CD154^+^ subsets were gated (blue, red and black frames, respectively) and further displayed as histograms according to TNF, IFN-γ and IL-2 expression. Frequencies among CD4^+^ T cells are indicated and color-coded on the histograms (markers are shown).
